# Designing cytochrome P450 enzymes for use in cancer gene therapy

**DOI:** 10.3389/fbioe.2024.1405466

**Published:** 2024-05-24

**Authors:** Saskya E. Carrera-Pacheco, Alexander Mueller, Juan A. Puente-Pineda, Johana Zúñiga-Miranda, Linda P. Guamán

**Affiliations:** Centro de Investigación Biomédica (CENBIO), Facultad de Ciencias de la Salud Eugenio Espejo, Universidad UTE, Quito, Ecuador

**Keywords:** genetic engineering, CYP enzymes, gene therapy, cancer, GDEPT, prodrug, P450

## Abstract

Cancer is a significant global socioeconomic burden, as millions of new cases and deaths occur annually. In 2020, almost 10 million cancer deaths were recorded worldwide. Advancements in cancer gene therapy have revolutionized the landscape of cancer treatment. An approach with promising potential for cancer gene therapy is introducing genes to cancer cells that encode for chemotherapy prodrug metabolizing enzymes, such as Cytochrome P450 (CYP) enzymes, which can contribute to the effective elimination of cancer cells. This can be achieved through gene-directed enzyme prodrug therapy (GDEPT). CYP enzymes can be genetically engineered to improve anticancer prodrug conversion to its active metabolites and to minimize chemotherapy side effects by reducing the prodrug dosage. Rational design, directed evolution, and phylogenetic methods are some approaches to developing tailored CYP enzymes for cancer therapy. Here, we provide a compilation of genetic modifications performed on CYP enzymes aiming to build highly efficient therapeutic genes capable of bio-activating different chemotherapeutic prodrugs. Additionally, this review summarizes promising preclinical and clinical trials highlighting engineered CYP enzymes’ potential in GDEPT. Finally, the challenges, limitations, and future directions of using CYP enzymes for GDEPT in cancer gene therapy are discussed.

## 1 Introduction

Gene therapy is a promising approach to correct gene mutations that cause genetic diseases, where the mutated gene is substituted with its normal version, usually through gene delivery to the affected cells via a viral vector (Zhao et al., 2022). The FDA recently approved several gene therapies that are successfully used to treat genetic diseases, including vision loss linked to congenital retinal dystrophy (Luxturna), hemophilia B (Hemgenix), beta-thalassemia (Zynteglo), and spinal muscular atrophy (Zolgensma) (Approved Cellular and Gene Therapy Products, 2023, https://www.fda.gov/vaccines-blood-biologics/cellular-gene-therapy-products/approved-cellular-and-gene-therapy-products).

Cancer is a substantial global socioeconomic burden, with millions of new cases and deaths occurring each year. In 2020, an estimated 19.3 million new cancer cases and almost 10 million cancer deaths occurred worldwide, making it the second most common cause of death (after heart disease), causing one in five deaths (Sung et al., 2021). The overall cost associated with cancer is a significant concern, with estimates projecting that the economic cost of cancers from 2020 to 2050 will exceed $25.2 trillion dollars globally (Chen et al., 2023). Therefore, strategic investments in cancer prevention and control measures, such as screening technologies and improved treatment options, are needed and could yield substantial health and economic benefits.

Unfortunately, the above-described approach for gene therapy is not as straightforward for cancer gene therapy since the high number and variations of genetic mutations in cancer cells, sometimes even within one type of cancer and within one patient, do not allow for such a “simple” gene therapy where only one gene needs to be replaced to cure the disease. Instead, multiple genetic targets need to be considered, e.g., oncogenes, tumor suppressor genes, suicide genes, immunomodulation approaches, expression of molecules affecting angiogenesis, tumor invasion, and metastasis (Seth, 2005).

Advancements in cancer gene therapy have revolutionized the cancer treatment landscape (Libutti, 2019; Cesur-Ergün and Demir-Dora, 2023). Understanding cancer as a disease mediated by somatic aberrations in the host genome has been a pivotal advancement in human genomics (Amer, 2014). This understanding has paved the way for developing gene therapy as a potential first-line treatment for neoplastic diseases (Das et al., 2015). The use of oncolytic viruses and bacteria, as well as the advances in genetic modification of cancer and immune cells, has led to numerous clinical trials for cancer therapy, with several progressing to late-stage product development (Husain et al., 2015; Cesur-Ergün and Demir-Dora, 2023). Furthermore, the emergence of precision medicine and the utilization of siRNA technology as a therapeutic modality for specific cancers, such as pancreatic cancer, showcase the diverse and targeted approaches explored in cancer gene therapy (Zorde Khvalevsky et al., 2013). However, it is important to note that these sophisticated cancer therapeutics may pose a high financial burden for patients, highlighting the societal challenge associated with addressing cancer (Advancing Cancer Therapy, 2021).

One promising approach to cancer gene therapy is to introduce genes that encode anticancer prodrug metabolizing enzymes like Cytochrome P450 (CYP) enzymes, which aid in the successful elimination of cancer cells (Waxman et al., 1999; Mishra et al., 2018). This approach is called gene-directed enzyme prodrug therapy (GDEPT) (Chen and Waxman, 2002). The introduced CYP enzymes can be genetically engineered to improve prodrug conversion to its active metabolites at pharmacologically relevant drug levels or to minimize chemotherapy side effects by improving the metabolism of toxic by-products of chemotherapy (Zanger and Schwab, 2013; Zhang et al., 2015). Additionally, the vehicles of the gene therapy (e.g., viral vectors) can be designed to target specific tissues, directing the CYP enzyme expression directly to the tumor cells where prodrug metabolism will be most effective and, at the same time, reducing toxic effects of chemotherapeutic drugs on healthy tissue (Wang and Yuan, 2006; Capasso et al., 2013; Sun et al., 2020).

This review highlights the importance of CYP enzymes in developing cancer gene therapies. It also summarizes the last advances and challenges in the genetic engineering of CYP enzymes with improved metabolic profiles of anti-cancer drugs for GDEPT.

## 2 Cytochrome P450 enzymes and cancer

### 2.1 Role of cytochrome P450 enzymes in drug metabolism

The substrate promiscuity these CYP enzymes exhibit makes them a key factor for studying drug interactions. These proteins are equipped with a prosthetic group composed of iron protoporphyrin IX, bound by a cysteine thiolate ligand at their active site (in most proteins). This active site is anchored in the molecule’s center, posing a challenge for immediate interaction with target ligands (Schenkman and Jansson, 2006; Urban et al., 2018). Furthermore, the catalytic cycle of CYP enzymes requires a multicomponent system for the transfer of a pair of electrons, facilitated by redox partners such as NADPH-cytochrome P450 oxidoreductase (CPR) and, less commonly, cytochrome b5 (Waskell and Kim, 2015; Jeffreys et al., 2018). Although the structure is highly similar across all families, ligand access channels can influence the enzyme’s selectivity for the substrate (Urban et al., 2018). Residues within these channels confer affinity to certain types of molecules, prioritizing them for the catalysis of reactions such as hydroxylation, epoxidation, deamination, and monooxygenation, among others (Zhao et al., 2021). An example of this is the N-hydroxylation generated in dapsone, an anti-leprosy medication, catalyzed by CYP enzymes 2B6, 2D6, 3A4, 2C8, 2C19, 2E1, 2C18, 2C9, or the oxygenation generated by 3A4 in desogestrel, which is a contraceptive medication (Rendic and Guengerich, 2021).

In metabolism, drugs typically undergo three main phases. The first phase aims to increase the molecule’s polarity through reactions such as oxidation, reduction, or hydrolysis (Conan et al., 2021). In these processes, CYP enzymes participate in 96% of the reactions, while the remaining percentage is distributed among enzymes such as aldo-keto reductase, microsomal flavin monooxygenase, and monoamine oxidase (Rendic and Guengerich, 2015). The second phase involves further increasing the polarity of molecules through conjugation reactions mediated by transferase enzymes such as N-acetyltransferases (NAT) and sulfotransferases (SULT), among others (Conan et al., 2021). Finally, the last phase consists of the excretion and elimination of metabolized compounds through transporters like P-glycoprotein (Xu et al., 2005). It is important to note that, in the first phase, CYP enzymes also play a role in the bioactivation of prodrugs. These compounds exhibit a therapeutically inactive conformation and require transformation to generate the desired effect (Rendic and Guengerich, 2021).

The CYP enzymes are responsible for metabolizing ∼75% of marketed drugs (Guengerich, 2010). Families 1 to 3 of these enzymes account for over 80% of the involvement in drug-associated metabolic reactions (Zhao et al., 2021). Additionally, the isoforms 3A4, 2C9, 2C19, 2D6, and 1A2 are responsible for ∼95% of drug oxidations (Guengerich, 2010).

An example of metabolic activation is acetaminophen, a commonly used analgesic and antipyretic, which is metabolized by the P450 isoforms 3A4, 2E1, 2D6, 1A2, and 2A6 (Rendic and Guengerich, 2021). However, these CYP enzymes are also involved in the bioactivation of various chemotherapeutic prodrugs used in cancer therapy.

### 2.2 Importance of CYP enzymes in cancer

Cytochrome P450 enzymes are closely linked to cancer, playing various roles, from the chemical transformation of antineoplastic drugs to acting as metabolizers of carcinogens (Elfaki et al., 2018; Zhao et al., 2021). Although it has been observed that isoforms 1A1, 1A2, 1B1, 2A6, 2A13, 2E1, and 3A4 are significantly involved in the metabolism of various carcinogens, there is no information on the percentage of involvement of these CYP isoforms in the biotransformation of carcinogens (Guengerich, 2010).

Most anticancer drugs undergo metabolism through various CYP enzymes isoforms. For example, tamoxifen, an estrogen receptor modulator with anticancer properties, is metabolized through different isoforms, mainly 2D6 and 3A5 (Goetz et al., 2005; Serrano et al., 2011). Another case is ellipticine, an antineoplastic agent and topoisomerase II inhibitor, metabolized through various isoforms such as 1A2, 1A1, 2C19, 2E1, 2D6, 2C9, 3A4, 2B6, and 1B1. In addition to its anticancer function, raloxifene prevents osteoporosis and is metabolized by CYP3A4 and CYP2D6 (Rendic and Guengerich, 2021). Similarly, oxazaphosphorines like cyclophosphamide (CPA) and ifosfamide (IFA) are activated by CYP enzymes. CYP2B6 is primarily responsible for CPA metabolism, while CYP3A4 is responsible for IFA metabolism. However, other isoforms, such as 2A6, 3A5, 2C9, 2C18, and 2C19, are also involved in the bioactivation of these drugs. CPA and IFA are used as chemotherapeutic agents in various cancer types, including breast, prostate, some lymphomas, and leukemia (Roy et al., 1999; Jounaidi and Waxman, 2004).

**FIGURE 1:**
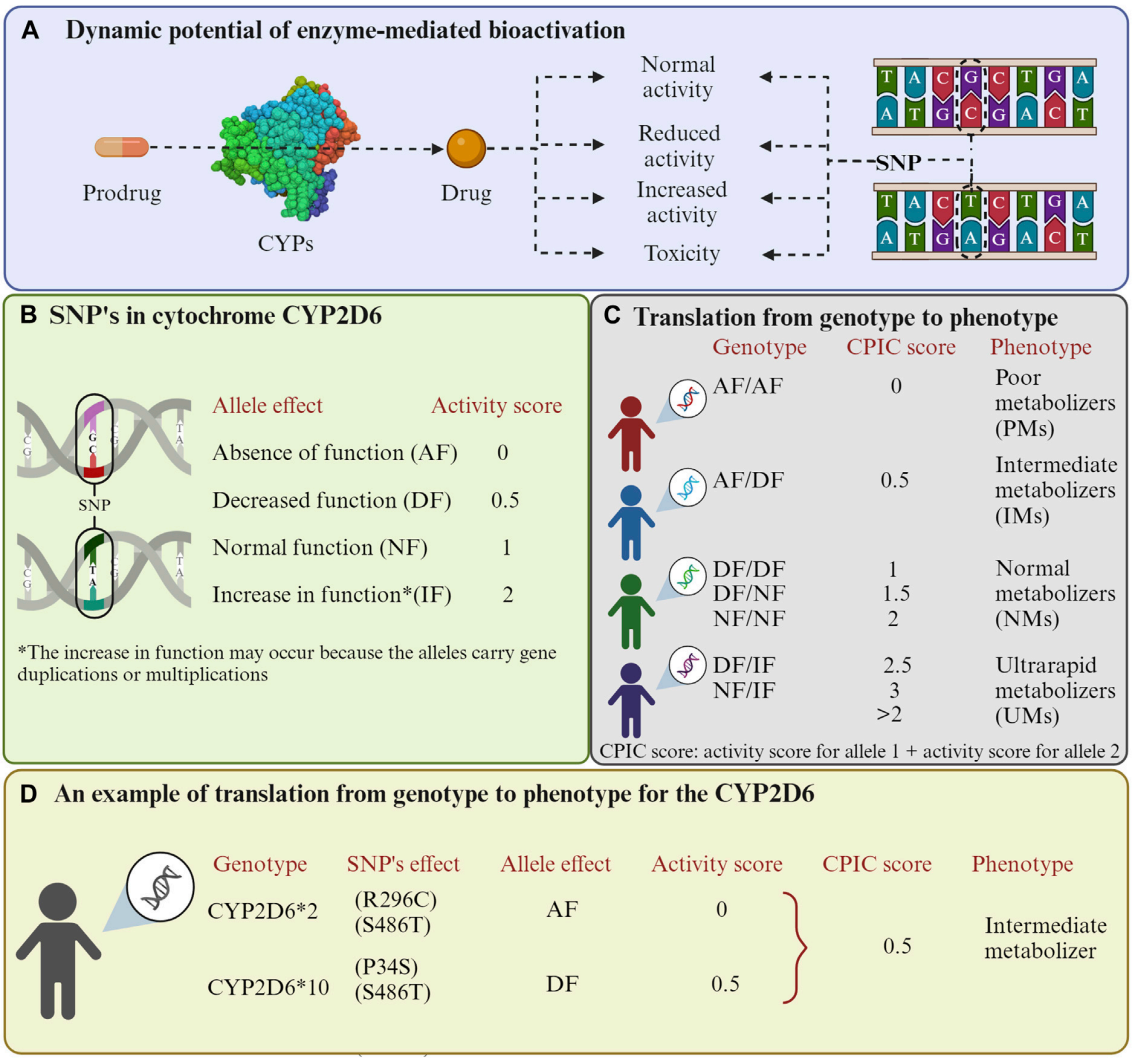
Enzyme-mediated bioactivation of prodrugs. **(A)** Enzyme-mediated bioactivation process. **(B)** Activation scores related to the effects that mutations in CYP2D6 can have. **(C)** Phenotype possibilities depending on the allele pair an individual possesses. The Clinical Pharmacogenetics Implementation Consortium (CPIC) method is used to classify phenotypes. **(D)** An example of numerical scores for the CYP2D6*2/10 polymorphisms are presented. It is important to highlight that cytochrome P450 enzymes exhibit variability in their substrates, so scores may differ depending on the drug. Additionally, other organizations such as the Dutch Pharmacogenetics Working Group (DPWG) may provide alternative phenotype classifications. The activation values were obtained from the study by (Gaedigk et al., 2008). The Figure was created using Biorender.com.

Nevertheless, enzyme-mediated bioactivation can lead to various possibilities. One possibility is that the prodrug may transform into its active form without changing, producing the desired effect. Another one is that the enzymes can cause an increase or loss of activity and even lead to toxicity for the individual (Guengerich, 2021). This last outcome is attributed to genetic polymorphisms (Gaedigk et al., 2018). In this context, individuals can exhibit different phenotypes based on the enzyme alleles they possess, and this allelic frequency varies among populations. Allelic variants of the enzyme have different affinities for the same substrate (Figure 1). The website Pharmacogene Variation Consortium (https://www.pharmvar.org/genes) gathers valuable information on the pharmacological interaction of various CYP polymorphisms.

Based on these polymorphisms, individuals have been classified as ultra-rapid metabolizer (UM), extensive metabolizer (EM), intermediate metabolizer (IM), and poor metabolizer (PM) (Mirabbasi et al., 2017; Caudle et al., 2020). This genetic diversity has a direct impact on how the body reacts to different medications, posing a challenge to healthcare as it can result in the lack of efficacy of treatments or the manifestation of adverse drug responses (Bosch et al., 2006). Due to the significant involvement of CYP enzymes in drug metabolism, it is crucial to identify their genetic peculiarities in an individual or population to optimize the efficacy and safety of pharmacological treatments (Goh et al., 2017). These CYP enzymes’ polymorphisms have been suggested to modulate the cancer risk of patients and contribute to individual susceptibility, particularly in the metabolism of tobacco-related compounds (Hernando-Rodriguez et al., 2012).

Among the CYP enzymes, 2D6, 2A6, and 2B6 show more polymorphisms in the Caucasian population and are closely related to drug metabolism (Preissner et al., 2013). For instance, the CYP2B6*6 allele, resulting from missense mutations (K262R, Q172H), leads to a decrease in its function due to its reduced expression in the liver (Ariyoshi et al., 2011; Preissner et al., 2013). However, the protein of the CYP2B6*6 allele shows a higher affinity for CPA, with a Km of 1.62 mM, compared to the normal allele, which has a Km of 2.68–4.03 mM (Ariyoshi et al., 2011; Lautier et al., 2016). In this case, the function depends more on the expression of the enzyme than on the affinity of a substrate. Conversely, the CYP1A2*1F allele with importance in processing antineoplastic drugs experiences an increase in its function; hence, individuals with the homozygous genotype (1*F/1*F) are classified as rapid metabolizers. Notably, the prevalence of this allele in European and American populations surpasses 60% (Neyshaburinezhad et al., 2021). An example where the activity is unaffected is the CYP2D6*2 allele, resulting from missense mutations (R296C, S486T), with more than a 30% allelic frequency in both the European and American populations (Neyshaburinezhad et al., 2021).

*In vivo* analysis of allelic variants provides relevant information for enzymatic engineering. Obtaining enzymes with improved characteristics opens the possibility of their use in gene therapy against cancer (i.e., GEDPT). This type of therapy offers particular benefits to individuals resistant to conventional cancer treatments, such as those with breast cancer carrying the CYP2C9*2 allele (R144C). This allele is associated with resistance to neoadjuvant chemotherapy, which is the initial step in treatment aimed primarily at halting tumor growth and reducing its size to facilitate surgical removal or improve the outcomes of radiotherapy in the future. The neoadjuvant chemotherapies utilize combinations of various drugs. For example, CMF includes CPA, methotrexate, and fluorouracil, while FAC comprises fluorouracil, doxorubicin, and CPA. The allelic frequency of CYP2C9*2 ranges between 6% and 13% in Europeans and Americans (Seredina et al., 2012; Neyshaburinezhad et al., 2021).

## 3 Cancer gene therapy

### 3.1 Principles of cancer gene therapy

Cancer gene therapy is a promising approach for treating cancer by introducing genetic material into cancer cells to fight the disease (Cross and Burmester, 2006). The key principle of cancer gene therapy involves delivering therapeutic genes into cancer cells or healthy tissue (e.g., immune cells) (Cesur-Ergün and Demir-Dora, 2023). Viral and non-viral vectors (e.g., liposomes and polymers) can carry the therapeutic genes and insert them into target cell DNA (Hwang et al., 2001; Sung and Kim, 2019; Manisha. B.; Shinde et al., 2020). Several approaches exist to achieve anti-cancer effects:

a. Gene therapy can replace mutated genes that cause or drive cancer growth and progression with normal functioning genes (gene correction, e.g., BRCA1 in breast cancer) (Obermiller et al., 2000).
b. Certain genes, like metastasis suppressor genes and tumor suppressor genes (e.g., BRMS1 and REIC/Dkk-3), can inhibit tumor growth and metastasis. Gene therapy can activate or overexpress these genes in cancer cells to suppress tumors (Campbell et al., 2000; Zhang et al., 2006; Smith et al., 2009; Su et al., 2015; Kalinichenko et al., 2017).
c. Oncogenes are genes that promote uncontrolled cell division (Yan et al., 2011). Gene therapy techniques can silence or inactivate oncogenes in cancer cells (Wendel et al., 2006; Poltronieri et al., 2013; Tatiparti et al., 2017).
d. Therapeutic genes and viral vectors can stimulate the body’s immune system to attack and eliminate cancer cells more effectively (Chiu et al., 2009; Shaw and Suzuki, 2019).
e. Gene therapy can modify cancer cells to reduce drug resistance, make them more sensitive to chemotherapy drugs, or increase enzymatic prodrug conversion, improving treatment outcomes (Chen and Waxman, 2002; Cammareri et al., 2010; Li et al., 2021). The goal of gene-directed enzyme prodrug therapy (GDEPT) with the design of CYP enzymes for cancer gene therapy is to leverage this principle, achieving more targeted and effective chemotherapy treatment while also aiming at reducing side effects from undesired toxic metabolites (Waxman et al., 1999; Zanger and Schwab, 2013; Zhang et al., 2015; Mishra et al., 2018).

This review further focuses on GDEPT, specifically the utilization of CYP enzymes therein, as a clever approach for cancer gene therapy. It introduces a group of well-studied enzymes and allows the use of already clinically approved drugs, aiming to improve their efficacy and specificity and reduce their side effects.

### 3.2 The role of CYP enzymes in gene-directed enzyme prodrug therapy (GDEPT)

**FIGURE 2:**
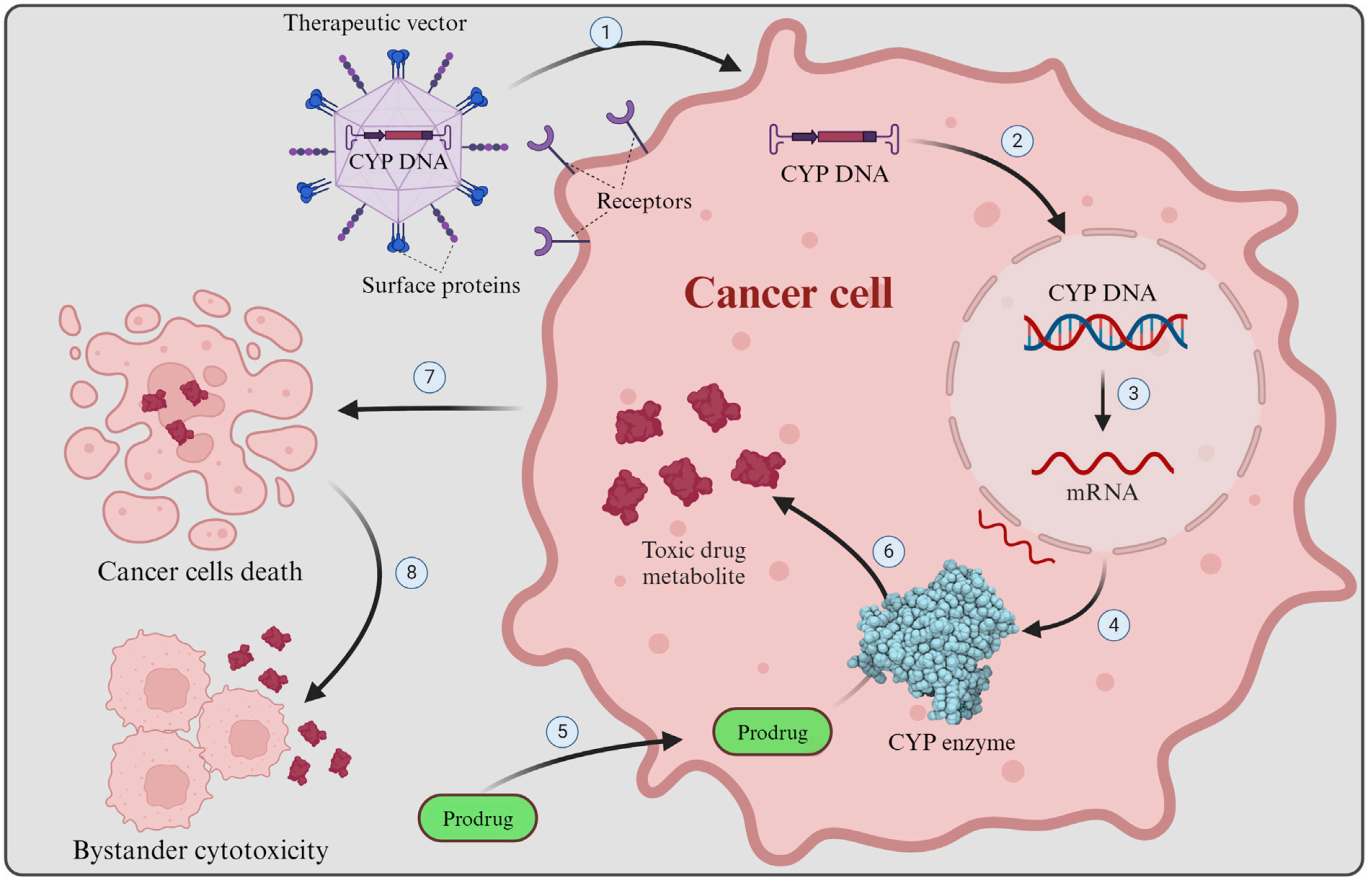
GDEPT in cancer therapy. GDEPT in cancer therapy is achieved by delivering enzymes (e.g., CYP) ideally directly to targeted tumor cells where non-toxic chemotherapy prodrugs are transformed by the introduced enzymes into their cytotoxic form to cause cancer cells death. 1) Receptor recognition results in viral entry into the targeted cancer cell, where the engineered CYP enzyme DNA is released. 2) The engineered DNA is inserted into the cancer cell nucleus and integrated into the host genome. 3) Transcription of the CYP DNA into mRNA. 4) CYP mRNA is translated into functional CYP enzymes. 5) Non-toxic chemotherapy prodrug enters the cancer cell. 6) The prodrug is metabolized by the engineered CYP enzymes into its active, cytotoxic form. 7) The cytotoxic drug metabolite causes damage to the cell (e.g., DNA damage), resulting in cancer cell death. 8) Upon cell death toxic drug metabolite is released and can affect nearby cancer cells, causing bystander cell cytotoxicity. The Figure was created using Biorender.com.

Gene-directed enzyme prodrug therapy (GDEPT) is a promising approach in cancer treatment, involving the delivery of a therapeutic gene encoding a foreign enzyme to tumor cells, where a systemically administered nontoxic chemotherapy prodrug can be converted into its active, cytotoxic metabolite upon expression of the enzyme, leading to cancer cell death (Figure 2) (Günther et al., 2006; Grohmann et al., 2009). Due to the selective genetic modification of tumor cells, this technique has shown potential to improve antitumor activity and selectivity for cancer cells of chemotherapy treatments (Mishra et al., 2018). GDEPT has been evaluated in various clinical trials using different enzyme/prodrug combinations, demonstrating its versatility and potential for clinical application (Wang et al., 2004; Hedley et al., 2007; Grohmann et al., 2009; Alekseenko et al., 2015). However, in terms of using GDEPT specifically for cancer treatment, sufficiently successful human clinical trials with the desired significant improvements are still lacking, despite promising results from preclinical studies as well as moderately successful early-stage clinical trials (Phases I and I/II, see Section 4.6) (Salmons et al., 2003; Braybrooke et al., 2005; Karjoo et al., 2016). Moreover, some studies have focused on optimizing GDEPT by incorporating novel enzymes and prodrugs, as well as improving the expression levels of prodrug-activating genes to enhance therapeutic efficacy (Chen and Waxman, 2002; Kratz et al., 2008; Gerth et al., 2019). Overall, GDEPT holds promise as a targeted and effective approach for cancer therapy, with ongoing research aiming to refine further and expand its applications.

The role of CYP enzymes in GDEPT is crucial for developing targeted cancer treatments. CYP enzymes, especially the CYP1, CYP2, and CYP3 families, metabolize endogenous and exogenous substances in the human body (Zanger and Schwab, 2013; Luo et al., 2021). They play a significant role in activating anticancer prodrugs within cancer cells, thereby converting non-cytotoxic prodrugs into cytotoxic drugs, which selectively target and kill cancer cells (Kumar, 2010). A wide range of clinically established anticancer drugs need these CYP enzymes to become activated to their cytotoxic form, with CYTOXAN (cyclophosphamide) and IFEX (ifosfamide) being thoroughly studied examples for use in GDEPT (Le Blanc and Waxman, 1989; Kivistö et al., 1995). Chen and Waxman (2002), Quiñones et al. (2008), provide a list of anti-cancer P450 prodrugs of interest for use in GDEPT. Furthermore, some commonly used chemotherapy drugs do not necessarily need the CYP enzymes to become active but still benefit from CYP metabolism by conversion into a more active metabolite. For example, CYP2B enzymes aid in the release of the cytotoxic aziridine moiety from Tepadina (Thiotepa), while CYP2D6 transforms the anti-estrogen breast cancer drug tamoxifen to the 100-fold more potent derivative 4-hydroxy-tamoxifen (Borgna and Rochefort, 1981; Waxman, 1993; Dehal and Kupfer, 1997).

However, for successful GDEPT in cancer patients, careful considerations must be considered. As mentioned before, multiallelic genetic polymorphisms in CYP enzymes influence the activity and function of these enzymes, leading to distinct pharmacogenetic phenotypes and, therefore, variations in the therapy responses of patients (Zhou et al., 2009; Zanger and Schwab, 2013). Furthermore, the modulation of CYP enzyme activity, e.g., by other drugs or dietary polyphenols, affects the pharmacokinetics and bioavailability of drugs, influencing the efficacy of chemotherapy, especially when combined with GDEPT (Korobkova, 2015).

GDEPT can be utilized to introduce desired CYP enzymes to achieve cell-specific gene delivery and expression, controlled conversion of prodrugs to drugs in target cells, and expanded toxicity to the target cells’ neighbors through bystander effects (Zhang et al., 2015). The bystander effect is crucial for GDEPT success because it removes the need to transduce all target tumor cells with the therapeutic gene, which is currently unattainable with existing gene delivery methods. The introduced CYP enzymes activate bioreductive cytotoxins, thereby increasing the efficacy of targeted therapy for drug-resistant hypoxic tumors (Pidkovka et al., 2021). Moreover, genetic engineering allows to develop and engineer optimized CYP enzymes with desired metabolic function and drug selectivity for use in GDEPT (Li et al., 2020). Importantly, the tumor cell-specific activation of selective CYP enzyme function through GDEPT allows for the use of lower doses of already approved chemotherapy prodrugs due to the increased sensitivity of the modified cells, potentially reducing toxicity on healthy tissue while maintaining therapeutic efficacy, highlighting the importance of this approach in cancer therapy (Miura et al., 2015; Mishra et al., 2018).

Moreover, the overexpression of certain CYPs, such as CYP1B1, in some cancer cells has led to the development of CYP inhibitors for chemoprevention as well as chemotherapy prodrugs designed to be activated by CYPs specifically expressed in cancer cells for cell-specific cytotoxic effects, further demonstrating the potential for targeted cancer treatments utilizing CYP enzymes (Bruno and Njar, 2007).

In summary, the role of CYP enzymes in GDEPT is pivotal for developing targeted and selective cancer therapies. The ability of CYP enzymes to activate prodrugs within cancer cells, the impact of genetic polymorphisms on CYP enzyme activity, and the potential to reduce toxicity while maintaining therapeutic efficacy highlight the significance of CYP enzymes in GDEPT. Developing engineered CYP enzymes for use in GDEPT represents a promising approach to cancer gene therapy.

## 4 Genetic engineering of cytochrome P450 enzymes

Genetic engineering of cytochrome P450 enzymes has emerged as a powerful tool in various fields, including organic synthesis, pharmaceutical development, and biotechnology. The primary reasons to engineer P450 enzymes include: i) enabling successful heterologous expression, ensuring high production yields, and enough enzyme quantities for further studies (Andersen and Møller, 2002; Jiang et al., 2021); ii) enhancing the solubility of the proteins, a necessary requirement for their crystallization and subsequent structure determination; iii) enhancing catalytic activity to improve the enzyme’s metabolic efficiency towards specific substrates or xenobiotics (Behera et al., 2010; Behrendorff et al., 2015); iv) optimizing the interactions between the CYP enzymes, their electron donors, and co-factors to favor activity and efficiency (Basudhar et al., 2015; Li et al., 2020; Zhang and Wang, 2022); v) improving robustness including stability, thermostability, and solvent tolerance for better performance under industrial conditions (Reinen et al., 2015; Gumulya et al., 2018; Harris et al., 2018); vi) enabling control over regioselectivity, stereoselectivity of their reactions for the synthesis of complex molecules (Zhang et al., 2011); and vii) expanding substrate recognition range (Butler et al., 2013; Li et al., 2020).

### 4.1 Design and optimization of CYP enzymes for cancer therapy

The design and optimization of CYP enzymes for cancer therapy requires a multidisciplinary approach, combining molecular biology, protein engineering, and pharmacology. As mentioned in Section 3, the genetic engineering of CYP enzymes has been explored for cancer therapy using the concept of GDEPT. In GDEPT, CYP enzymes activate prodrugs selectively within tumor cells, leading to localized cytotoxic effects. The ultimate goal is to develop CYP enzymes with improved expression, stability, catalytic activity, regioselectivity, and tumor-specific delivery to enhance the effectiveness of cancer treatment while minimizing side effects.

**FIGURE 3:**
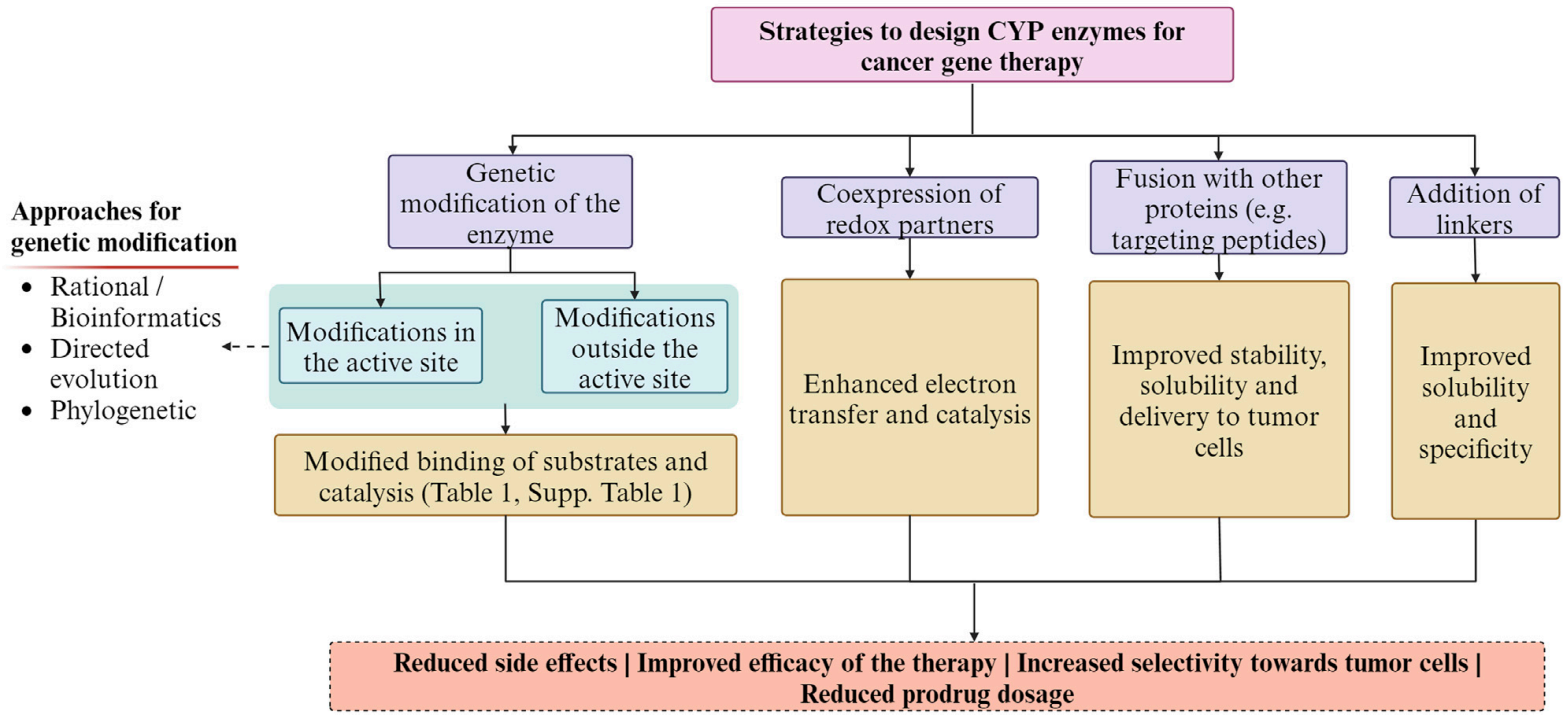
Summary of the strategies employed for designing CYP enzymes for cancer gene therapy and the expected outcomes. The Figure was created using Biorender.com.

CYP enzymes can have multiple potential sites of metabolism on a given substrate molecule. One strategy is optimizing their regioselectivity to selectively target specific regions of a prodrug or anticancer agent to improve the efficacy and selectivity of cancer therapy (Sun et al., 2007). Importantly, the recognition of substrates, their access to the active site, and the binding of redox partners are not solely influenced by the residues within the active site but also by residues outside of it (Kumar et al., 2005; Sun et al., 2007), and should be considered during the engineering of these enzymes. Additionally, optimizing the catalytic activity and modifying the structure of prodrugs or anticancer agents could enhance substrate binding and catalysis and their specificity towards CYP enzymes (Chen et al., 2004; Jounaidi et al., 2006; Chen et al., 2007; Sun et al., 2007). A way to improve its catalytic activity is the co-expression of CYP reductases to allow proper electron transfer (Lengler et al., 2006; Touati et al., 2014; Sellner et al., 2021). Fusion of CYP enzymes with other proteins, such as reductases or targeting peptides, can enhance their stability, solubility, and specific delivery to tumor cells, improving the therapeutic potential for cancer therapy (Steffens et al., 2000; Kan et al., 2001; Jounaidi and Waxman, 2004; Tychopoulos et al., 2005; Jounaidi et al., 2006). Other recently explored alternatives are the addition of a detachable linker, which breaks down within the tumor microenvironment, and the addition of a functional carrier to the original anticancer drug that could help the release of a molecule that has enhanced pharmacokinetic and pharmacological properties at the tumor site, transforming traditional antineoplastic agents into prodrugs (Najjar and Karaman, 2019). Figure 3, summarizes some of the strategies employed and the expected outcomes.

### 4.2 Approaches for genetic modification of CYP enzymes for cancer therapy

Several methods and techniques for engineering CYP enzymes have been employed together or separately over the past decades, yielding enzymes with improved metabolic efficiency toward specific anticancer drugs (Table 1, Supplementary Table S1). Nevertheless, other approaches are becoming new alternatives to produce robust, more stable, and novel substrate affinity CYP enzymes. The next paragraphs describe these alternatives:

**TABLE 1:** Summary of the most relevant mutations or changes produced in different CYP enzymes’ studies for use in cancer gene therapy.

CYP name	Mutations or changes	Reaction	Km (mM)	Vmax (mol/min/mol)	Vmax/Km (mol/min/mol P450/ mM)	Kcat (min^-1^)	Kcat/Km (min^-1^ mM^-1^)	SA (pmol product/min/mg enzyme)	KD (µM)	Conver- sion (µM)	Prodrug	Approach	Ref
2B1	WT	4-hydroxylation	1.45	35.9	24.9	N.D.	N.D.	N.D.	N.D.	N.D.	CPA	Rational/ Site-directed mutagenesis	(Chen et al. 2004)
	I114V		0.4	20	50	N.D.	N.D.	N.D.	N.D.	N.D.			
	WT		1.73	13.2	7.9	N.D.	N.D.	N.D.	N.D.	N.D.	IFA		
	V363A		1	10	9	N.D.	N.D.	N.D.	N.D.	N.D.			
	2B1dH (N-terminal modified)		∼0.4	N.D.	N.D.	∼34	∼75	N.D.	N.D.	N.D.	CPA	Directed evolution	(Kumar et al. 2005)
	L209A, S334P		∼0.2	N.D.	N.D.	∼50	∼220	N.D.	N.D.	N.D.			
	2B1dH (N-terminal modified)		∼0.5	N.D.	N.D.	∼10	∼22	N.D.	N.D.	N.D.	IFA		
	L209A, V183L		∼0.3	N.D.	N.D.	∼24	∼70	N.D.	N.D.	N.D.			
2B6	WT		4.9	62.5	12.8	N.D.	N.D.	N.D.	N.D.	N.D.	CPA	Rational/ Site-directed mutagenesis, molecular dynamics	(Nguyen et al. 2008)
	I114V/ V477W		1.1	58.5	52.7	N.D.	N.D.	N.D.	N.D.	N.D.			
2B6TM	L199M, I114V, V477W		1.05	105.5	100.5	N.D.	N.D.	N.D.	N.D.	N.D.	CPA	Rational/ Site-directed mutagenesis	(Touati et al. 2014)
2B11	WT		0.16	28.2	174.7	28	175	N.D.	N.D.	N.D.	CPA	Rational/ Site-directed mutagenesis	(Sun et al. 2007;Chen et al. 2004)
	2B11dH V183L		0.06	N.D.	N.D.	∼24	∼400	N.D.	N.D.	N.D.			
	WT		0.08	5.3	66.8	5.4	54	N.D.	N.D.	N.D.	IFA		
	2B11dH V183L		0.03	N.D.	N.D.	∼2.8	∼93	N.D.	N.D.	N.D.			
2B6 and 2B11	2B6 WT		4.03	N.D.	N.D.	N.D.	N.D.	23.2	N.D.	N.D.	CPA	Semi-rational/combinatorial approach of protein quantitative structure–activity relationships (QSAR)	(Lautier et al. 2016)
	2B11 WT		0.08	N.D.	N.D.	N.D.	N.D.	13.2	N.D.	N.D.			
	Chim K		0.07	N.D.	N.D.	N.D.	N.D.	8.3	N.D.	N.D.			
	Chim O		0.02	N.D.	N.D.	N.D.	N.D.	11.8	N.D.	N.D.			
BM3 (CYP102A1)	R47L, E64G, F81I, F87V, E143G, L188Q, Y198C, E267V, H285Y, G415S **(M11)**		0.087- 0.16	896- 1614	10315- 10078	N.D.	N.D.	N.D.	N.D.	N.D.	CPA	Rational/ Site-directed mutagenesis and random mutagenesis	(Vredenburg et al. 2015; van Vugt-Lussenburg et al. 2007; Damsten et al. 2008)
	R47L, E64G, F81I, F87V, E143G, L188Q, Y198C, E267V, H285Y, G415S, L437S		0.088- 0.115	757-938	8620- 8129	N.D.	N.D.	N.D.	N.D.	N.D.	CPA		
	R47L, E64G, F81I, F87V, E143G, L188Q, Y198C, E267V, H285Y, G415S, L437S		N.D.	N.D.	577- 580.9	N.D.	N.D.	N.D.	N.D.	N.D.	IFA		
	M11		N.D.	N.D.	1306- 1430	N.D.	N.D.	N.D.	N.D.	N.D.	IFA		
4B1	WT	Furan ring epoxidation	N.D.	N.D.	N.D.	N.D.	N.D.	N.D.	30	16.8	4-IPO	Rational/Homology model, mutagenesis	(Wiek et al. 2015)
	S427P, R124K, E130D, E159D, R199K, T202S, D217E, L135F, V156I, L226I, T158A, E170K, N190D **(hP427+12)**		N.D.	N.D.	N.D.	N.D.	N.D.	N.D.	28	11.6			
	S427R		N.D.	N.D.	N.D.	N.D.	N.D.	N.D.	8	5.1		N.A.	(Roellecke et al. 2017)
	hP427+12		N.D.	N.D.	N.D.	N.D.	N.D.	N.D.	1.31	150.9	PK		
	S427R		N.D.	N.D.	N.D.	N.D.	N.D.	N.D.	0.25	42.3			
1A1	E161K	N-demethylation	0.249	30	120.5	N.D.	N.D.	N.D.	N.D.		DTIC	Rational/Homology model, mutagenesis	(Lewis et al. 2011)
	V228T		0.386	32.4	83.9	N.D.	N.D.	N.D.	N.D.				
	E256K		0.238	29	121.8	N.D.	N.D.	N.D.	N.D.				

CPA, cyclophosphamide; IFA: ifosfamde, 4-IPO, 4-ipomeanol; DTIC, dacarbazine; PK, perilla ketone; N.D., Not determined; WT, wild type.

#### 4.2.1 Rational and bioinformatics approach

Rational design involves the use of site-directed or random mutagenesis. It aims to improve the protein’s features by enhancing hydrophobic core packing, establishing salt bridges, incorporating disulfide bonds, substituting glycine, introducing proline residues, and shortening protein loops (Thomson et al., 2022). However, this approach relies on a detailed understanding of the protein structure, good-quality datasets, and bioinformatics tools to predict the effects of specific amino acid substitutions or modifications in the CYP enzyme. Tools such as molecular cloning, molecular docking, and molecular dynamics are frequently used with other strategies to engineer P450 enzymes. Artificial intelligence methods are anticipated to significantly transform our prediction and comprehension of protein structure and stability.

Most genetic engineering efforts to design CYP enzymes for cancer therapy have focused on using site-directed mutagenesis in combination with molecular modeling (Table 1, Supplementary Table S1). For instance, an N-terminal modified version of the enzyme CYP2B11 (2B11dH) replaced F202L, I209A, and V183L residues, increasing the metabolism of the anti-cancer prodrugs CPA and IFA in the V183L mutant (Sun et al., 2007). The mutant showed a 2.7-fold reduction in Km for CPA and IFA 4-hydroxylation compared to the wild type. Nguyen et al. (2008), produced five mutants of CYP2B6, which displayed a catalytic efficiency that was 2–3 times higher, and by combining the two most successful mutations, a double mutant achieved a 4-fold enhancement in Km/Vmax. Another study reported two CYP BM3 mutants carrying 11 and 12 substitutions, showing the fastest reported CPA and IFA 4-hydroxylation rates (∼10,315 and ∼1306 mol/min/mol P450/mM, respectively) to date (Vredenburg et al., 2015).

In another strategy, Lautier et al. (2016), used sequence element swaps to build chimeras between the CYP2B11 enzyme in dogs and the CYP2B6 enzyme in humans. Given the better affinity of the canine enzyme for CPA, with a Km of 0.08 mM, analogous segments of each enzyme were exchanged progressively in the experiment. As a result, the chimera 2BchO (ChimO) was obtained, demonstrating the best affinity for CPA, with a 4-fold enhancement in Km (Lautier et al., 2016) (Table 1, Supplementary Table S1). Additionally, using homology models, CYP1A1 and CYP4B1 mutants attained catalytically enhanced dacarbazine (DTIC) and 4-ipomeanol (4-IPO) activation, respectively (Table 1, Supplementary Table S1) (Lewis et al., 2011; Wiek et al., 2015).

#### 4.2.2 Directed evolution

This process involves the iterative cycles of random mutagenesis, recombination, and screening, mimicking the principles of natural evolution in a laboratory setting (Arnold, 1996). This iterative process of mutation and selection allows for the evolution of CYP enzymes with improved activity, stability, or other desired traits. This approach could convert the P450 promiscuous generalist enzymes into specialists capable of mediating reactions of interest with exquisite regio- and stereo-selectivity (Behrendorff et al., 2015). One drawback is that great screening efforts are necessary to identify beneficial mutants. Therefore, developing a time-effective, cheap, and efficient high-throughput screening system is imperative to select mutants with desired traits (Kumar, 2010). For instance, Copp et al. (2014), developed a screening platform that enables the directed evolution of any prodrug-activating enzymes utilizing an inducible SOS promoter fused to a modified GFP reporter gene. This allows the assessment of DNA damage levels within intact *Escherichia coli* through fluorescence-activated cell sorting (FACS). Remarkably, this study achieved a significant 90,000-fold enrichment of a functional prodrug-activating nitroreductase from a background library with no activity (Copp et al., 2014).

In contrast to the rational design, neither protein’s structural information nor computational tools are needed (Encell et al., 1999). However, computational methods (e.g., SCHEMA) have been utilized to enhance the effectiveness of directed evolution methods, similar to rational design (Otey et al., 2004).

Even though directed evolution has shown outstanding results in the protein engineering field, this approach has only been used in P450-based cancer gene therapy by (Kumar et al., 2005). In this study, it was used to create a N-terminal modified 2B1dH double mutant V183L/L209A, which exhibited enhanced catalytic efficiency (kcat/Km) in activating IFA, while L209A/S334P showed improved kcat/Km in activating CPA (Table 1, Supplementary Table S1). These changes equated to 6-fold improvement in the Km value for CPA and IFA 4-hydroxylation compared to wild-type CYP2B1 (Kumar et al., 2005).

#### 4.2.3 Phylogenetic methods

Phylogenetic methods such as the consensus approach and the ancestral sequence reconstruction (ASR) have been used to engineer proteins (Amin et al., 2004; Bershtein et al., 2008; Gumulya et al., 2018; Harris et al., 2022). The consensus approach generates a consensus sequence by aligning and comparing related protein sequences. The consensus sequence represents the shared characteristics of the related proteins and can then be used as a template for designing new proteins with improved properties. On the other hand, ASR is a computational method used to reconstruct the amino acid sequence of an ancient protein, typically by analyzing the sequences of its modern descendants. This reconstructed protein could show beneficial and unique traits useful for diverse applications (Spence et al., 2021).

One common feature of these approaches is using a dataset of protein sequences to generate multiple sequence alignment (MSA) files to identify conserved regions and variable regions. ASR, additionally, will generate a phylogenetic tree that is used to infer the evolutionary history of the protein and estimate the most likely sequence of the ancestral protein at each branch point of the tree (Thomson et al., 2022). Opposite to the previous approaches, both phylogenetic strategies benefit from a reduced mutant screening effort; however, their drawbacks include, among others, the uncertainty in the consensus sequence or the evolutionary history of the protein (Aadland and Kolaczkowski, 2020; Spence et al., 2021; Thomson et al., 2022).

Although these approaches have enabled high production yields, improved stability, novel activity, and substrate specificity for diverse CYP enzymes, to the best of our knowledge, they have not been exploited within the cancer gene therapy field. One strategy could include the resurrection of CYP1 to CYP4 families to obtain novel or similar substrate affinities. For instance, CYP1A1, 2B6, 2B11, 3A4, or 4B1, can be resurrected with improved activity, expression yields, and stability.

Notably, ASR has already been applied to engineer gene therapy viral vectors, which are also critical to developing efficient cancer gene therapy treatments (Zinn et al., 2015; Xiao et al., 2016).

### 4.3 Challenges in genetic engineering of CYP enzymes for cancer gene therapy

Some challenges of CYP enzyme’s genetic engineering include achieving successful and efficient expression, as these enzymes are often membrane-bound proteins that require specific conditions and co-expression of other proteins, such as redox partners, to be active (Lengler et al., 2006). Efficient electron transfer from redox partners could require the construction of linkers between them to facilitate their interactions (Touati et al., 2014).

An alternative under exploration is introducing non-human redox partners for P450 enzymes in gene therapy. CYP enzymes can be reconstituted with surrogate redox partners, enabling electron transfer during catalysis. For instance, a study by Liu X. et al. (2022) compared three pairs of frequently-used surrogate redox partners: Fdx1499/FdR0978, Adx/AdR, and Pdx/PdR, and found that Fdx1499/FdR0978 showed the most promise in terms of electron transfer properties (Liu X. et al., 2022). Moreover, the biological diversity of P450 redox partner systems is vast, and several new types of P450 redox partner systems have been characterized (Sadeghi and Gilardi, 2013; McLean et al., 2015). This suggests that there is potential for using alternative redox partners to modulate the catalytic activity of P450s, which could be beneficial for gene therapy applications. However, non-human redox partners must be compatible with human cells and not elicit an immune response. Moreover, producing non-human redox partners in human cells can be difficult, and ensuring their stability within the cellular environment adds another layer of complexity. It is important to carefully evaluate the potential benefits of combining cytochrome P450 and redox partner *in vivo* models that are relevant to the specific tissue being treated, as the effect of redox partner on the activity of CYP enzymes seems to differ depending on the type of cell being used (Lengler et al., 2006). Alternatively, “Molecular Lego” has been used to engineer catalytically self-sufficient CYP enzymes (Dodhia et al., 2006). This modular assembly of different protein domains could create chimeric proteins with desired properties. For instance, the human P450 domain has been fused with a non-human redox partner (*Bacillus megaterium* reductase-BMR), creating a single polypeptide chain that functions effectively within human cells (Gilardi et al., 2002; Fairhead et al., 2005; Dodhia et al., 2006; Catucci et al., 2022).

Moreover, several studies described the development of fusion proteins that combine CYP enzymes with other enzymes, such as monomeric sarcosine oxidase (CYP152B1-polyG-MSOX fusion protein) (Giuriato et al., 2022) or to promote CYP enzymes’ peroxygenase catalysis (CYP102A1, CYP152B1) (Paul et al., 2014; Shoji et al., 2016; Hardiyanti Oktavia et al., 2023) to enable P450-catalyzed reactions without the need for an external electron donor. These recent developments in P450 fusion proteins represent significant progress toward creating efficient and versatile biocatalysts for various applications, including gene therapy. Nevertheless, it is important to overcome the limitations of using an *in situ* H_2_O_2_ generation approach, such as potential cytotoxicity due to reactive oxygen species, lack of specificity in targeting cancer cells, and challenges in controlling H_2_O_2_ levels effectively.

Another challenge is finding appropriate enzyme-specific promoters, which will only drive expression in the target tissue, which is also a limitation in other GDEPTs (Robson and Hirst, 2003). The “Molecular Lego” approach could also aid designing expression vectors that allow for the modular assembly of genetic elements, such as promoters, enhancers, coding sequences for P450 enzymes, and targeting sequences. This modular design could enable the precise control of CYP enzyme expression levels, tissue specificity, and subcellular localization, optimizing therapeutic efficacy and minimizing off-target effects (Dodhia et al., 2006).

CYP enzymes exhibit a wide range of substrate specificities, and this specificity is not only given by the interaction between residues in the catalytic site but also by residues outside it (Kumar et al., 2005). Therefore, it can be challenging to engineer them to accept new substrates or alter their selectivity towards specific compounds. The successful modification will usually require a combination of engineering methods. Additionally, since they are promiscuous enzymes, they could show unwanted interactions with other drugs, affecting the therapy efficacy or creating unpredicted side effects (Ekroos and Sjögren, 2006; Nath et al., 2010).

**FIGURE 4:**
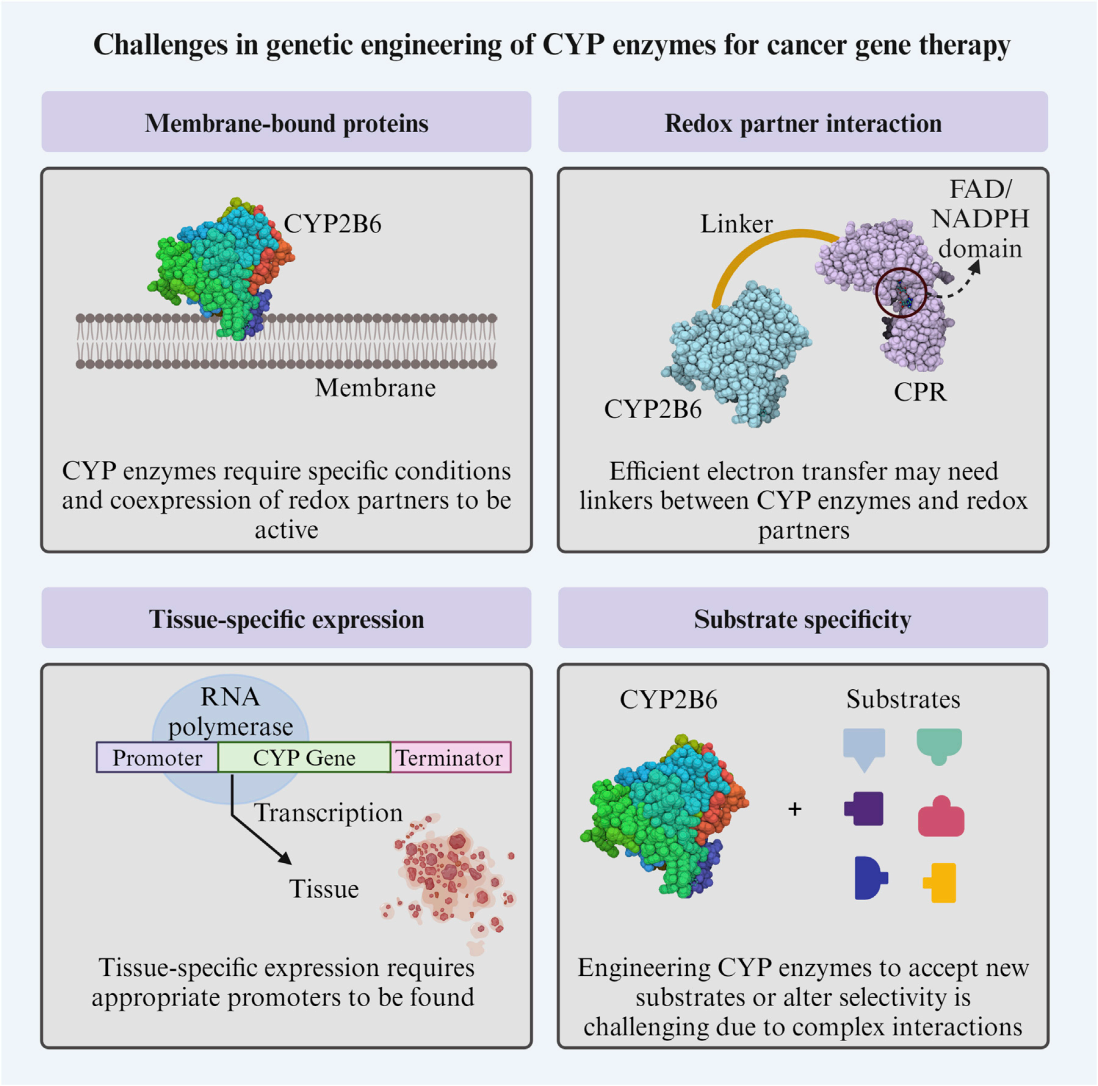
Challenges in genetic engineering of CYP enzymes for cancer gene therapy. CYP: cytochrome P450; CPR: cytochrome P450 reductase. The Figure was created using Biorender.com.

These challenges (Figure 4) highlight the complexity of engineering CYP enzymes and the need for a multidisciplinary approach combining molecular biology, biochemistry, and computational methods.

### 4.4 Strategies to minimize off-target effects

The design and optimization of CYP enzymes for cancer therapy often involve strategies to minimize off-target effects and enhance tumor-specific activation of prodrugs.

GDEPT involves the delivery of CYP enzymes and their redox partners directly to tumor cells using gene therapy approaches. This reduces the side effects usually seen in the systemic delivery of prodrugs, which are activated by the CYP enzymes in the liver. Furthermore, the use of drug-metabolizing enzymes that are intrinsic to the tumor could mediate the local generation of cytotoxins, eliminating the need for complex delivery systems (e.g., CYP4B1 predominantly expressed in lung cells) (Travica et al., 2013). Another strategy is the coexpression of CYP enzymes with their redox partners in tumor cells to enhance tumor-specific activation of prodrugs (Tychopoulos et al., 2005; Mahato et al., 2011). CYP enzymes can be fused to targeting peptides to improve their tumor-specific delivery, as seen in other GDEPTs (Liu X. et al., 2022), and can also benefit from utilizing viral or non-viral vectors to deliver CYP enzymes selectively to tumor cells (Quester et al., 2017; Tapia-Moreno et al., 2017). Additionally, the “Molecular Lego” approach has the potential to enhance the accuracy of controlling the levels of CYP enzyme expression, as well as its specificity to certain tissues and its location within cells. By doing so, it can optimize the effectiveness of therapeutic treatments while reducing any unintended side effects (Dodhia et al., 2006).

### 4.5 Promising results and case studies

CYP enzymes have gained attention in cancer therapy due to their potential role in targeted drug delivery and treatment. While this field of research is still evolving, there have been some promising results in their use for cancer therapy and their engineering for the same purpose. Table 1; Supplementary Table S1 summarize the genetic engineering efforts for obtaining CYP enzymes with desirable gene therapy features.

#### 4.5.1 CYP2B family and cyclophosphamide (CPA)/ifosfamide (IFA)

Some members of the CYP2B family can activate the prodrugs CPA and IFA used in chemotherapy and therefore have been the target for cancer therapy research. Two metabolic pathways are prevalent for these prodrugs, with the 4-hydroxylation pathway resulting in the DNA-alkylating phosphoramide mustard (active cytotoxic metabolite) and acrolein (Huang et al., 2000b). However, the undesired N-dechloroethylation pathway yields the nephro- and neurotoxic metabolites dechloroethyl-CPA/IFA and chloroacetaldehyde (Huang et al., 2000b). Genetic engineering of CYP enzymes can result in more efficient prodrug metabolism and in enzymes that favor desired metabolic pathways over undesired ones to reduce side effects (Patterson and Murray, 2002; Nguyen et al., 2008; Kumar, 2010).

It was identified that rat CYP2B1 exhibited a significantly higher efficiency in catalyzing CPA 4-hydroxylation compared to rabbit CYP2B4 or 2B5, with a 10 to 35-fold increase. By replacing several residues in CYP2B1, the efficiency of CPA and IFA 4-hydroxylation was significantly improved (Chen et al., 2004). CYP2B1 was used in clinical trials together with IFA (see section 4.6). Furthermore, the canine CYP2B11 demonstrated 7 to 8 times greater activity as a CPA and IFA 4-hydroxylase than CYP2B1 (Chen et al., 2004). Using directed evolution on residues that are far from the active site, the catalytic efficiency of CYP2B1 was improved by 2.8-fold of kcat/Km for CPA and 3.5-fold of kcat/Km for IFA (Kumar et al., 2005). In another study, CYP2B11 was used in mice with gliosarcomas, where CPA was directly injected into the tumor, and its release into the bloodstream was slowed down by utilizing the slow-release polymer poloxamer 407 as a carrier for delivering CPA. This resulted in a total of 3.9-fold increase in intratumoral and in antitumor activity (Chen et al., 2007). Subsequently, CYP2B11 was re-engineered for improved CPA and IFA metabolism by introducing six different mutations at the N-terminal (P450 2B11dH). These changes yielded enhanced catalytic efficiency for both substrates (Sun et al., 2007) (Table 1, Supplementary Table S1). In the past, chimeras of CYP2B5, CYP2B4, and CYP2B11 have been created to investigate the metabolism of different substrates (Kedzie et al., 1993; Szklarz et al., 1996); however, in 2016, Lautier and colleagues used this approach to create fifteen chimeras between CYP2B6 and CYP2B11 with the aim of improving CPA affinity. From these, chimeras K and O showed the lowest Km values, providing insights into the structural elements that control CPA specificity in these enzymes (Lautier et al., 2016).

In another study, some human CYP2B6 residues were replaced by the specific amino acids found in the substrate recognition sequences of CYP2B11. As a result, a double mutant (Table 1; Supplementary Table S1) showed a 4-fold increase in Km/Vmax. It transformed a resistant human head and neck cancer cell line (A-253) into a sensitive cell line towards CPA, unlike the wild-type CYP2B6 (Nguyen et al., 2008).

A fusion protein CYP2B6-NADPH cytochrome P450 reductase (RED) proved to be effective in enhancing the cytotoxicity of CPA in pulmonary tumor cell lines with low levels of endogenous RED, following infection and treatment (Tychopoulos et al., 2005). Another fusion protein consisting of a triple mutant of CYP2B6 (Table 1; Supplementary Table S1) and RED (CYP2B6TM-RED) was introduced in resistant human (A549) and murine (TC1) pulmonary cell lines by a recombinant lentivirus vector and showed successful transformation of these into cell lines susceptible to CPA (Touati et al., 2014).

A study explored using a CYP2B6 gene therapy in combination with neural stem/progenitor cells for treating glioblastoma multiforme (GBM), a highly aggressive brain tumor. The results showed substantial impairment of tumor growth upon CPA administration (Mercapide et al., 2010).

#### 4.5.2 CYPBM3 and CPA/IFA

CYPBM3 (CYP102) mutants obtained by Vredenburg et al. (2015) showed the fastest rate of CPA and IFA 4-hydroxylation reported to date (Table 1; Supplementary Table S1). However, they have not yet been tested in tumor cell lines or animal models. CYPBM3 is catalytically self-sufficient, eliminating the need for a redox partner (Vredenburg et al., 2015). Notably, CYPBM3 is of bacterial origin, and immunogenicity challenges could limit its application in cancer gene therapy. However, functional and structural insights for designing and developing new CYP enzymes can be obtained through these studies.

#### 4.5.3 CYP4B1 and 4-ipomeanol (4-IPO)

CYP4B1 catalyzes the oxidative metabolism of 4-IPO (furan ring epoxidation), leading to the formation of reactive intermediates. These electrophilic species have demonstrated cytotoxic effects, suggesting a potential role in cancer treatment (Parkinson et al., 2012). Re-engineering of the human CYP4B1 enzyme based on the rabbit homolog CYP4B1 yielded an efficient activator of 4-IPO in HepG2 human hepatoma cells (Table 1; Supplementary Table S1), making it a candidate for liver and lung cancer therapy. Importantly, exchange of S427P in the human 4B1 alone is critical for partially restoring catalytic activity against 4-IPO. However, it does not alone control the functional activity of the enzyme; therefore, additional mutations are needed to fine-tune its activity (Zheng et al., 2003).

In a study by Roellecke et al. (2017), human (h-P427) and rabbit (r-P422) CYP4B1 as well as the human mutant (h-P + 12) were compared for their affinity (KD) and conversion against different substrates (Table 1 and Supplementary Table S1). The substrates were structurally related and were tested for cytotoxic activity in human liver-derived cells. Interestingly, h-P + 12 showed very high conversion values for perilla ketone (PK), a strong cytotoxin for HepG2 cells, making this a promising prodrug candidate for suicide gene therapy enzymes (Roellecke et al., 2017). Further studies were performed by Kowalski et al. (2019) to identify new prodrugs for CYP4B1 with improved and desired features for gene therapy. The findings provide insights on how to better design prodrugs highly activated by CYP4B1 (Kowalski et al., 2019).

#### 4.5.4 CYP1A1 and dacarbazine (DTIC)

CYP1A1 is involved in the metabolic activation of DTIC through N-demethylation, producing the active metabolite 5-aminoimidazole-4-carboxamide (AIC). AIC further undergoes spontaneous degradation to form diazomethane, a reactive intermediate responsible for the alkylating effects on DNA. This DNA alkylation contributes to the cytotoxic properties of DTIC, making it a valuable chemotherapeutic agent in the treatment of certain cancers. Importantly, DTIC is metabolized in the liver by CYP1A1, CYP1A2, and CYP2E1 (Reid et al., 1999). Mutants E161K, V228T and E256K showed a 1.7-fold increase in catalytic efficiency (Vmax/Km) for DTIC N-demethylation (Table 1; Supplementary Table S1).

### 4.6 Pre- and clinical trials

Preclinical studies have been conducted to evaluate GDEPT, including the involvement of cytochrome P450 enzymes, as demonstrated by a study conducted by McErlane and colleagues in 2005. In this study, the enzyme CYP2B6 was utilized in combination with radiation, along with the prodrugs banoxantrone (AQ4N) and CPA. DNA damage was observed in RIF-1 cells transfected with the enzyme and treated with AQ4N, supporting its ability to metabolize the drug. The antitumor capacity of the treatment was confirmed in a murine RIF-1 tumor model. Additionally, it was highlighted that the same gene can function effectively in oxygen-rich environments and under hypoxic conditions, as CPA requires oxygen for its toxic activity, unlike AQ4N, which can achieve the same goal in hypoxic conditions (McErlane et al., 2005).

Regarding clinical trials, promising results have been observed, as seen in the phase I-II study conducted by Löhr and colleagues in 2003. In this study, 14 patients with inoperable stage III-IV pancreatic adenocarcinoma were treated, and patients who had previously undergone chemotherapy were not accepted. Procedures involved the intra-arterial administration of microencapsulated cells with CYP2B1, followed by the application of ifosfamide. The results showed no toxicity greater than grade II in any 14 patients. Throughout the study, no increase in tumor size was observed in any participants (stable disease), and two exhibited a tumor volume reduction exceeding 50% (partial response). These findings indicate favorable treatment tolerance and suggest a positive impact on reducing tumor burden in certain individuals (Löhr et al., 2003).

Another phase I/II clinical trial employed a retroviral vector MetXia-P450 to induce the expression of CYP2B6. This approach aimed to transfect cancer cells, thereby generating enzyme expression and activating CPA to trigger its toxic effect in the affected cells. The study involved 9 patients with advanced breast cancer skin nodules and 3 patients with melanoma, who received two intratumoral injections of MetXia-P450. In some cases, the results revealed a partial response, with four patients maintaining a stable disease condition while the rest experienced disease progression. Although ten out of twelve participants showed positive transfection control, it is important to note that transduction affected less than 1% of tumor cells in this specific trial (Hunt, 2001; Braybrooke et al., 2005). Due to the very low transduction percentage, progress to other phases of clinical studies has not been made.

As of the writing date of this manuscript, no clinical trials beyond those mentioned in this article have been identified. This absence could be attributed to the focus of applying this therapy primarily to treating the primary tumor, with less attention given to metastasis. However, it is important to note that this therapeutic modality could be integrated with other anticancer treatments, thereby expanding its applicability and effectiveness in the comprehensive approach to cancer therapy.

## 5 Challenges and limitations

**FIGURE 5:**
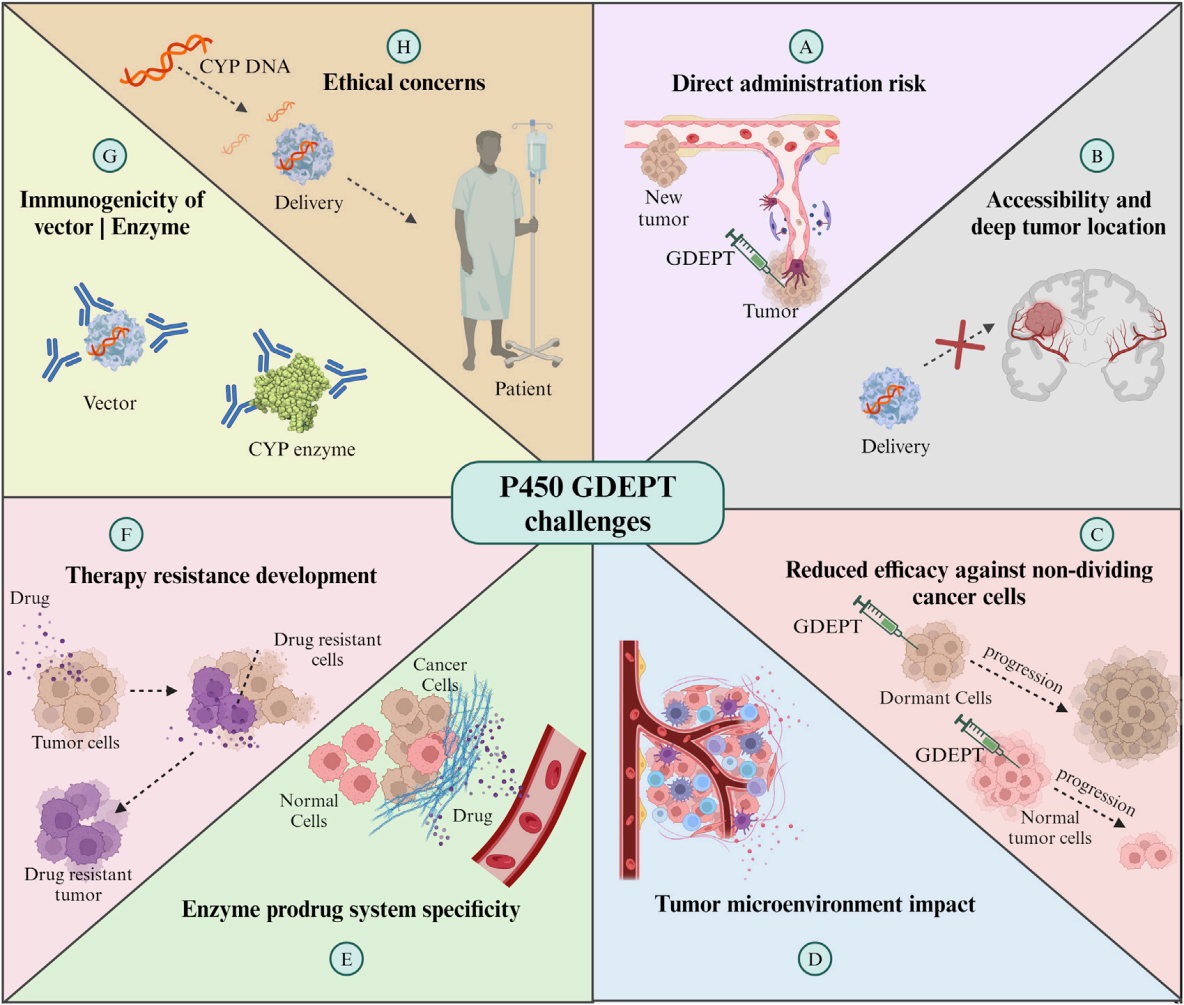
Challenges of CYP enzymes-GDEPT in cancer treatment. For further details refer to the text. GDEPT: gene-directed enzyme prodrug therapy. The Figure was created using Biorender.com.

GDEPT is an innovative cancer treatment strategy. Efficiency in the delivery of therapeutic genes and local intratumoral activation of prodrugs present promising treatment methods, minimizing systemic side effects. However, the effectiveness of these approaches faces significant challenges (Figure 5). On the one hand, gene delivery must overcome physical barriers and the immune response to reach and be adequately expressed in a sufficient number of tumor cells, which is crucial for the effective activation of the prodrug (Zhao et al., 2021). For example, the low expression and activity of CYPs may reduce the activation of antitumor agents in tumor cells, whilst the overexpression of CYPs in tumor cells may rapidly devitalize tumor agent substrates, which may be associated with treatment resistance and cause subsequent tumor relapse (Verma et al., 2019).

On the other hand, direct administration of vectors for expression in the tumor area, although it reduces systemic exposure to chemotherapeutic agents, involves risks such as bleeding and seeding of metastatic cells. Additionally, the administration technique is limited by the distribution of the vector along the needle tracts with incomplete coverage of the entire tumor nodule, reducing the effectiveness of the therapy (Braybrooke et al., 2005). Furthermore, direct administration is limited to accessible tumors, as mentioned by Braybrooke et al. (2005) in their study, CYP2B6 gene was delivered in a total of nine patients with breast cancer and three with melanoma by using human CYP2B6 commercial retroviral vector, MetXia and cyclophosphamide, which showed promising results but limited use for the treatment of systemic metastases (Braybrooke et al., 2005). The deep location of some types of tumors can complicate direct and repeated injections into the tumor area. Endoscopic delivery emerges as an alternative, although limited by pancreatic duct obstruction in most tumors (Löhr et al., 2003). In 2003, a study employed a different and safe delivery method. Angiography was used to position capsules containing genetically modified allogeneic cells directly into an artery that feeds the tumor. These cells were engineered to express the enzyme CYP2B1, which activates IFA. The local activation of IFA at the tumor site allowed for concentrated drug therapy with reduced systemic toxicity, potentially increasing the efficacy of the treatment. The results indicated a higher survival rate for patients treated with this method compared to those receiving conventional treatments (Löhr et al., 2003). Although this study managed to overcome the administration barrier, issues with micrometastasis still remain a challenge for GDEPT therapy. This outlook underscores the need to optimize therapeutic gene delivery and expression strategies while exploring safe and effective delivery methods to maximize therapeutic benefits in cancer treatment.

The reduced efficacy against non-dividing cancer cells is another significant limitation. Many cancer treatments, including enzyme and prodrug systems, are most effective against rapidly dividing cells (Ortiz de Montellano, 2013). However, in every tumor, there is a population of cells that divide slowly or are in a resting state. These cells are less susceptible to being affected by the therapy, which may contribute to treatment resistance and cancer recurrence. Therefore, the development of strategies that can also effectively target these non-active cells is essential to improve therapeutic outcomes in enzyme- and prodrug-based treatments.

Another significant challenge is the specificity of the enzyme-prodrug system. As mentioned in previous sections, the inherent promiscuity of enzymes such as CYPs, which allows them to interact with a wide range of substrates, poses a significant challenge in redesigning them for selective prodrug activation. The enzyme expressed by the therapeutic gene should ideally only activate the prodrug at the tumor site. However, if the enzyme is expressed in non-target tissues or if the activated prodrug can diffuse out of the tumor, this can cause damage to healthy cells and result in various clinical complications, such as systemic toxicity or damage to specific organs (McFadyen et al., 2004). Furthermore, the tumor microenvironment itself can influence the efficacy of GDEPT. Factors like hypoxia, heterogeneous blood supply, and the presence of various cytokines (IL-1, IL-6, TNF-α, and IFN-γ) and enzymes can affect the activity or expression level of the therapeutic enzyme cytochrome P450, the distribution of the prodrug and its metabolites within the tumor (Fradette and Du Souich, 2004; Stipp and Acco, 2021).

Additionally, the development of resistance to therapy is a concern. Tumors can evolve mechanisms to inactivate the therapeutic enzyme or efflux the activated drug, diminishing the effectiveness of the treatment (McFadyen et al., 2004). Finally, the potential immunogenicity of the vector or the therapeutic enzyme represents a challenge, as it could lead to an immune response that reduces the therapy’s effectiveness and causes harm to the patient (Stipp and Acco, 2021). These challenges necessitate ongoing research and development to refine GDEPT strategies, enhance their specificity and efficacy, and ensure their safety in clinical applications.

Finally, genetic engineering is raising ethical concerns in human patients, as the long-term effects remain unknown, off-target mutations and other edits can be made to the genome, and any changes can be heritable. Regulatory bodies require extensive preclinical and clinical data to show that the therapies are safe and effective, which is time-consuming and expensive.

## 6 Future directions

**FIGURE 6:**
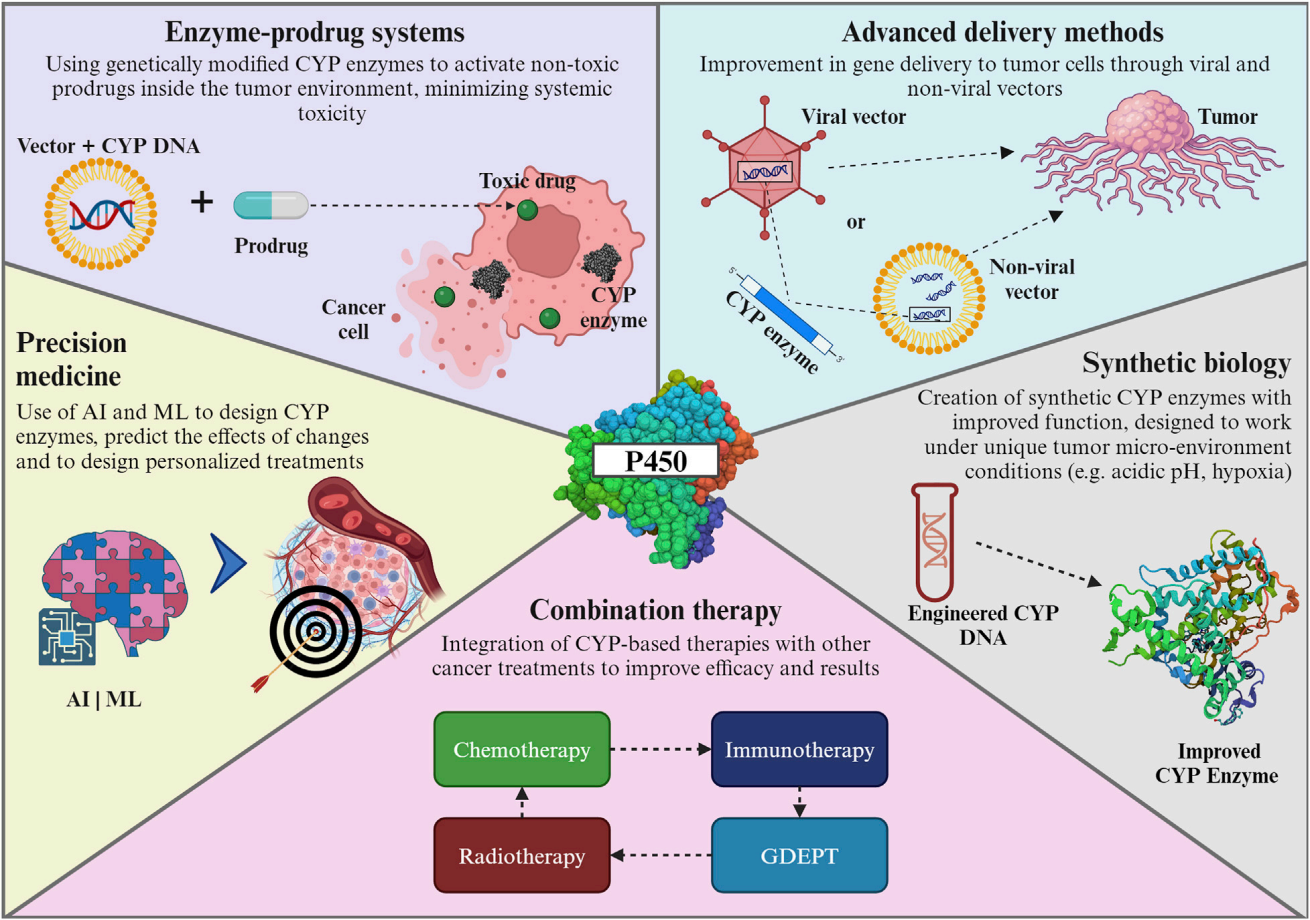
Emerging trends in genetic engineering of CYP enzymes. For further details refer to the text. CYP: cytochrome P450; AI: Artificial intelligence; ML: Machine learning; GDEPT: gene-directed enzyme prodrug therapy. The Figure was created using Biorender.com.

Emerging trends in the genetic engineering of CYP enzymes for cancer gene therapy focus on developing targeted and efficient treatments. Key advancements are summarized in Figure 6 and include: i) enzyme-prodrug systems utilizing engineered cytochrome P450 enzymes to activate non-toxic prodrugs within the tumor environment, minimizing systemic toxicity and enhancing treatment specificity (Malekshah et al., 2016). These systems utilize genetically engineered enzymes, such as modified versions of cytochrome P450, to activate non-toxic prodrugs specifically within the tumor environment (Ono et al., 2021). This approach minimizes systemic toxicity and enhances the therapeutic index of cancer treatments (Karjoo et al., 2016); ii) advanced delivery vectors to improve the delivery of therapeutic genes to tumor cells using optimized viral and non-viral vectors enhancing the efficiency and specificity of gene therapy (Naso et al., 2017; Wang et al., 2019). The exploration of non-viral methods such as lipid nanoparticles, has expanded the toolkit for delivering gene therapies. This progress is crucial for the effective application of cytochrome P450-based therapies, ensuring that the therapeutic genes reach their target cells with precision (Ediriweera et al., 2021); iii) the use of synthetic biology to create synthetic CYP enzymes with enhanced functionalities tailored for the tumor microenvironment, the ability to design enzymes that can work under the unique conditions present in tumor tissues, such as hypoxia or acidic pH, opens up possibilities for treating a broader range of cancers more effectively (McIntosh et al., 2014; Ja et al., 2016); and iv) a combination of therapies, integrating cytochrome P450-based therapies with other cancer treatments like immunotherapy or chemotherapy, could improve the overall treatment efficacy and patient outcomes (Huang et al., 2000a; Günzburg and Salmons, 2005; Jounaidi et al., 2006; Gomez et al., 2010).

The forefront of these trends is marked by a concerted effort to develop targeted therapies that precisely attack cancer cells while sparing healthy tissues, a paradigm shift from traditional, less discriminative treatments (Zhang et al., 2012). A major innovation combines insights from various scientific disciplines - such as bioinformatics, synthetic biology, and pharmacogenomics - to create more effective cytochrome P450-based therapies (Ozdemir et al., 2006). This holistic approach leverages the strengths of each field, leading to more robust and innovative treatment strategies.

The role of artificial intelligence (AI) and machine learning (ML) in the design of cytochrome P450 enzymes for cancer therapy is becoming increasingly pivotal, heralding a new era of precision medicine (Wang et al., 2022; Guengerich, 2023). Their ability to analyze vast datasets, predict outcomes, and design personalized treatments is transforming cancer therapy into a more effective and patient-centric approach. For example, AI and ML algorithms are adept at predicting how modifications in enzyme structures could affect their function (Li et al., 2023). This predictive capability is crucial for designing enzymes that are more efficient and specific in targeting cancer cells; AI can also help to accelerate enzyme modification by rapidly prototyping various enzyme configurations, thereby speeding up the discovery and development of effective cancer therapies (Hasegawa et al., 2010; Goldwaser et al., 2022). Finally, AI algorithms can integrate genetic, clinical, and pharmacological data to design personalized enzyme therapies. Tailoring treatments to individual patient profiles can achieve higher efficacy and lower toxicity (Squassina et al., 2015; van der Lee et al., 2020; Rao, 2023).

## 7 Conclusion

In the realm of cancer gene therapy, various strategies have been explored, including the transfer of tumor suppressor genes, suicide genes, enzyme/pro-drug approach (like GDEPT), inhibition of dominant oncogenes, immunomodulation approaches, and expression of molecules affecting angiogenesis, tumor invasion, and metastasis (Seth, 2005). The use of recombinant adeno-associated viruses (rAAV2) for intraperitoneal gene delivery to cancer cells, besides bacteria-mediated cancer gene therapy and the development of new vector systems, exemplifies the continuous progress and advanced strategies being employed in this field (Cao et al., 2010; Malecki et al., 2010; Zu and Gao, 2021; Bulcha et al., 2021; Thoidingjam et al., 2023). Of utmost importance remains bridging the gap between research and clinical application for successful clinical translation of recent advances.

The progress in cancer gene therapy has not been without challenges. Regulatory considerations for approval, viral shedding after gene therapy, and tissue-specific expression of suicide genes are among the critical aspects that necessitate thorough evaluation and understanding (Liu et al., 2006; Kawahira et al., 2010; Husain et al., 2015). Furthermore, the attitude of oncology physicians and nurses toward the acceptance of new drugs for gene therapy and the need for clinical trial optimization and pharmacogenomics underscore the importance of addressing not only the scientific and technical aspects but also the social and ethical dimensions of cancer gene therapy (Curiel et al., 2000; Liu et al., 2011; Baianu, 2012; Ginn et al., 2018; Kassi and Stoner, 2019).

CYP enzymes are the main enzymes responsible for drug metabolism. Therefore, a thorough understanding of each isoform’s interaction with different substrates and their accessibility to the catalytic center is important for their pharmacological application. Genetic engineering of CYP enzymes has employed a multidisciplinary strategy, combining molecular biology, protein engineering, and pharmacology, to address challenges in optimizing CYP enzymes for cancer therapy, specifically GDEPT. Key strategies involve enhancing enzyme expression, stability, catalytic activity, regioselectivity, and tumor-specific delivery. Techniques such as rational design, directed evolution, and phylogenetic methods contribute to developing tailored CYP enzymes that selectively activate prodrugs within tumor cells. Promising preclinical and clinical trials highlight the potential of engineered CYP enzymes in targeted cancer treatment despite ongoing challenges related to gene delivery, efficient expression, and minimizing off-target effects. However, it remains essential to refine GDEPT’s application further and ensure its safety and efficacy in clinical use.

In conclusion, the future of cancer gene therapy via cytochrome P450 enzyme engineering is marked by innovative approaches to enhance treatment specificity and efficiency. Advancements such as enzyme-prodrug systems, improved delivery vectors, the application of synthetic biology, and the integration with other therapies are steering the field towards more targeted and less toxic cancer treatments. The evolution of these technologies, coupled with the synergistic use of artificial intelligence and machine learning, is not only improving the design and delivery of these therapies but also paving the way for precision medicine in oncology. By leveraging these cutting-edge techniques, researchers are moving closer to developing therapies that can effectively target cancer cells while minimizing harm to healthy tissues, ultimately aiming to transform cancer treatment into a more effective, patient-centric approach.

## References

[B1] Aadland K. Kolaczkowski B. (2020). Alignment-integrated reconstruction of ancestral sequences improves accuracy. Genome Biol. Evol. 12, 1549–1565. 10.1093/gbe/evaa164 32785673 PMC7523730

[B2] Advancing Cancer Therapy (2021). Advancing cancer therapy. Nat. Cancer 2, 245–246. 10.1038/s43018-021-00192-x 35121963

[B3] Alekseenko I. V. Snezhkov E. V. Chernov I. P. Pleshkan V. V. Potapov V. K. Sass A. V. (2015). Therapeutic properties of a vector carrying the HSV thymidine kinase and GM-CSF genes and delivered as a complex with a cationic copolymer. J. Transl. Med. 13, 78. 10.1186/s12967-015-0433-0 25880666 PMC4359447

[B4] Amer M. H. (2014). Gene therapy for cancer: present status and future perspective. Mol. Cell. Ther. 2, 27. 10.1186/2052-8426-2-27 26056594 PMC4452068

[B5] Amin N. Liu A. D. Ramer S. Aehle W. Meijer D. Metin M. (2004). Construction of stabilized proteins by combinatorial consensus mutagenesis. Protein Eng. Des. Sel. 17, 787–793. 10.1093/protein/gzh091 15574484

[B36] Andersen M. D. Møller B. L. (2002). Use of methylotropic yeast Pichia pastoris for expression of cytochromes P450. Methods Enzymol. 357, 333–342. 10.1016/S0076-6879(02)57691-6 12424923

[B6] Approved Cellular and Gene Therapy Products (2023). Approved cellular and gene therapy products. Available at: https://www.fda.gov/vaccines-blood-biologics/cellular-gene-therapy-products/approved-cellular-and-gene-therapy-products (Accessed November 30, 2023).

[B7] Ariyoshi N. Ohara M. Kaneko M. Afuso S. Kumamoto T. Nakamura H. (2011). Q172H replacement overcomes effects on the metabolism of cyclophosphamide and efavirenz caused by CYP2B6 variant with Arg262. Drug Metab. Dispos. 39, 2045–2048. 10.1124/dmd.111.039586 21821736

[B8] Arnold F. H. (1996). Directed evolution: creating biocatalysts for the future. Chem. Eng. Sci. 51, 5091–5102. 10.1016/S0009-2509(96)00288-6

[B9] Baianu I. (2012). Cancer clinical trials optimization and pharmacogenomics. Nat. Preced. 10.1038/npre.2012.7046.1

[B10] Basudhar D. Madrona Y. Kandel S. Lampe J. N. Nishida C. R. de Montellano P. R. O. (2015). Analysis of cytochrome P450 CYP119 ligand-dependent conformational dynamics by two-dimensional NMR and X-ray crystallography. J. Biol. Chem. 290, 10000–10017. 10.1074/jbc.M114.627935 25670859 PMC4400317

[B11] Behera R. K. Goyal S. Mazumdar S. (2010). Modification of the heme active site to increase the peroxidase activity of thermophilic cytochrome P450: a rational approach. J. Inorg. Biochem. 104, 1185–1194. 10.1016/j.jinorgbio.2010.07.008 20709408

[B12] Behrendorff J. B. Y. H. Huang W. Gillam E. M. J. (2015). Directed evolution of cytochrome P450 enzymes for biocatalysis: exploiting the catalytic versatility of enzymes with relaxed substrate specificity. Biochem. J. 467, 1–15. 10.1042/BJ20141493 25793416

[B13] Bershtein S. Goldin K. Tawfik D. S. (2008). Intense neutral drifts yield robust and evolvable consensus proteins. J. Mol. Biol. 379, 1029–1044. 10.1016/j.jmb.2008.04.024 18495157

[B14] Borgna J. L. Rochefort H. (1981). Hydroxylated metabolites of tamoxifen are formed *in vivo* and bound to estrogen receptor in target tissues. J. Biol. Chem. 256, 859–868. 10.1016/s0021-9258(19)70058-1 7451477

[B15] Bosch T. M. Meijerman I. Beijnen J. H. Schellens J. H. M. (2006). Genetic polymorphisms of drug-metabolising enzymes and drug transporters in the chemotherapeutic treatment of cancer. Clin. Pharmacokinet. 45, 253–285. 10.2165/00003088-200645030-00003 16509759

[B16] Braybrooke J. P. Slade A. Deplanque G. Harrop R. Madhusudan S. Forster M. D. (2005). Phase I study of MetXia-P450 gene therapy and oral cyclophosphamide for patients with advanced breast cancer or melanoma. Clin. Cancer Res. 11, 1512–1520. 10.1158/1078-0432.CCR-04-0155 15746054

[B17] Bruno R. D. Njar V. C. O. (2007). Targeting cytochrome P450 enzymes: a new approach in anticancer drug development. ChemInform 38. 10.1002/chin.200739257 PMC1958998 17544277

[B18] Bulcha J. T. Wang Y. Ma H. Tai P. W. L. Gao G. (2021). Viral vector platforms within the gene therapy landscape. Signal Transduct. Target. Ther. 6, 53. 10.1038/s41392-021-00487-6 33558455 PMC7868676

[B19] Butler C. F. Peet C. Mason A. E. Voice M. W. Leys D. Munro A. W. (2013). Key mutations alter the cytochrome P450 BM3 conformational landscape and remove inherent substrate bias. J. Biol. Chem. 288, 25387–25399. 10.1074/jbc.M113.479717 23828198 PMC3757202

[B20] Cammareri P. Scopelliti A. Todaro M. Eterno V. Francescangeli F. Moyer M. P. (2010). Aurora-a is essential for the tumorigenic capacity and chemoresistance of colorectal cancer stem cells. Cancer Res. 70, 4655–4665. 10.1158/0008-5472.CAN-09-3953 20460511

[B21] Campbell I. Magliocco A. Moyana T. Zheng C. Xiang J. (2000). Adenovirus-mediated p16INK4 gene transfer significantly suppresses human breast cancer growth. Cancer Gene Ther. 7, 1270–1278. 10.1038/sj.cgt.7700226 11023200

[B22] Cao S. Cripps A. Wei M. Q. (2010). New strategies for cancer gene therapy: progress and opportunities. Clin. Exp. Pharmacol. Physiol. 37, 108–114. 10.1111/j.1440-1681.2009.05268.x 19671071

[B23] Capasso C. Hirvinen M. Cerullo V. (2013). Beyond gene delivery: strategies to engineer the surfaces of viral vectors. Biomedicines 1, 3–16. 10.3390/biomedicines1010003 28548054 PMC5423465

[B24] Catucci G. Ciaramella A. Di Nardo G. Zhang C. Castrignanò S. Gilardi G. (2022). Molecular lego of human cytochrome P450: the key role of heme domain flexibility for the activity of the chimeric proteins. Int. J. Mol. Sci. 23, 3618. 10.3390/ijms23073618 35408976 PMC8998974

[B25] Caudle K. E. Sangkuhl K. Whirl-Carrillo M. Swen J. J. Haidar C. E. Klein T. E. (2020). Standardizing *<scp>CYP</scp>2D6* genotype to phenotype translation: consensus recommendations from the clinical pharmacogenetics implementation consortium and Dutch pharmacogenetics working group. Clin. Transl. Sci. 13, 116–124. 10.1111/cts.12692 31647186 PMC6951851

[B26] Cesur-Ergün B. Demir-Dora D. (2023). Gene therapy in cancer. J. Gene Med. 25, e3550. 10.1002/jgm.3550 37354071

[B27] Chen C. S. Jounaidi Y. Su T. Waxman D. J. (2007). Enhancement of intratumoral cyclophosphamide pharmacokinetics and antitumor activity in a P450 2B11-based cancer gene therapy model. Cancer Gene Ther. 14, 935–944. 10.1038/sj.cgt.7701092 17853921 PMC2613860

[B28] Chen C.-S. Lin J. T. Goss K. A. He Y. Halpert J. R. Waxman D. J. (2004). Activation of the anticancer prodrugs cyclophosphamide and ifosfamide: identification of cytochrome P450 2B enzymes and site-specific mutants with improved enzyme kinetics. Mol. Pharmacol. 65, 1278–1285. 10.1124/mol.65.5.1278 15102956

[B29] Chen L. Waxman D. J. (2002). Cytochrome P450 gene-directed enzyme prodrug therapy (GDEPT) for cancer. Curr. Pharm. Des. 8, 1405–1416. 10.2174/1381612023394566 12052216

[B30] Chen S. Cao Z. Prettner K. Kuhn M. Yang J. Jiao L. (2023). Estimates and projections of the global economic cost of 29 cancers in 204 countries and territories from 2020 to 2050. JAMA Oncol. 9, 465–472. 10.1001/jamaoncol.2022.7826 36821107 PMC9951101

[B31] Chiu T.-L. Lin S.-Z. Hsieh W.-H. Peng C.-W. (2009). AAV2-mediated interleukin-12 in the treatment of malignant brain tumors through activation of NK cells. Int. J. Oncol. 35, 1361–1367. 10.3892/ijo_00000454 19885559

[B32] Conan M. Théret N. Langouet S. Siegel A. (2021). Constructing xenobiotic maps of metabolism to predict enzymes catalyzing metabolites capable of binding to DNA. BMC Bioinforma. 22, 450. 10.1186/s12859-021-04363-6 PMC8454073 34548010

[B33] Copp J. N. Williams E. M. Rich M. H. Patterson A. V. Smaill J. B. Ackerley D. F. (2014). Toward a high-throughput screening platform for directed evolution of enzymes that activate genotoxic prodrugs. Protein Eng. Des. Sel. 27, 399–403. 10.1093/protein/gzu025 24996412

[B34] Cross D. Burmester J. K. (2006). Gene therapy for cancer treatment: past, present and future. Clin. Med. Res. 4, 218–227. 10.3121/cmr.4.3.218 16988102 PMC1570487

[B35] Curiel D. T. Gerritsen W. R. Krul M. R. L. (2000). Progress in cancer gene therapy. Cancer Gene Ther. 7, 1197–1199. 10.1038/sj.cgt.7700222 10975681

[B37] Damsten M. C. van Vugt-Lussenburg B. M. A. Zeldenthuis T. de Vlieger J. S. B. Commandeur J. N. M. Vermeulen N. P. E. (2008). Application of drug metabolising mutants of cytochrome P450 BM3 (CYP102A1) as biocatalysts for the generation of reactive metabolites. Chem. Biol. Interact. 171, 96–107. 10.1016/j.cbi.2007.09.007 17996858

[B38] Das S. K. Menezes M. E. Bhatia S. Wang X.-Y. Emdad L. Sarkar D. (2015). Gene therapies for cancer: strategies, challenges and successes. J. Cell. Physiol. 230, 259–271. 10.1002/jcp.24791 25196387 PMC4363073

[B39] Dehal S. S. Kupfer D. (1997). CYP2D6 catalyzes tamoxifen 4-hydroxylation in human liver. Cancer Res. 57 (16), 3402–3406. 9270005

[B41] Dodhia V. R. Fantuzzi A. Gilardi G. (2006). Engineering human cytochrome P450 enzymes into catalytically self-sufficient chimeras using molecular Lego. J. Biol. Inorg. Chem. 11, 903–916. 10.1007/s00775-006-0144-3 16862439

[B42] Ediriweera G. R. Chen L. Yerbury J. J. Thurecht K. J. Vine K. L. (2021). Non-viral vector-mediated gene therapy for ALS: challenges and future perspectives. Mol. Pharm. 18, 2142–2160. 10.1021/acs.molpharmaceut.1c00297 34010004

[B43] Ekroos M. Sjögren T. (2006). Structural basis for ligand promiscuity in cytochrome P450 3A4. Proc. Natl. Acad. Sci. U. S. A. 103, 13682–13687. 10.1073/pnas.0603236103 16954191 PMC1564212

[B44] Elfaki I. Mir R. Almutairi F. M. Duhier F. M. A. (2018). Cytochrome P450: polymorphisms and roles in cancer, diabetes and atherosclerosis. Asian pac. J. Cancer Prev. 19, 2057–2070. 10.22034/APJCP.2018.19.8.2057 30139042 PMC6171375

[B45] Encell L. P. Landis D. M. Loeb L. A. (1999). Improving enzymes for cancer gene therapy. Nat. Biotechnol. 17, 143–147. 10.1038/6142 10052349

[B46] Fairhead M. Giannini S. Gillam E. M. J. Gilardi G. (2005). Functional characterisation of an engineered multidomain human P450 2E1 by molecular Lego. J. Biol. Inorg. Chem. 10, 842–853. 10.1007/s00775-005-0033-1 16283395

[B47] Fradette C. Du Souich P. (2004). Effect of hypoxia on cytochrome P450 activity and expression. Curr. Drug Metab. 5, 257–271. 10.2174/1389200043335577 15180495

[B48] Gaedigk A. Ingelman-Sundberg M. Miller N. A. Leeder J. S. Whirl-Carrillo M. Klein T. E. (2018). The pharmacogene variation (pharmvar) consortium: incorporation of the human cytochrome P450 (CYP) allele nomenclature database. Clin. Pharmacol. Ther. 103, 399–401. 10.1002/cpt.910 29134625 PMC5836850

[B49] Gaedigk A. Simon S. D. Pearce R. E. Bradford L. D. Kennedy M. J. Leeder J. S. (2008). The CYP2D6 activity score: translating genotype information into a qualitative measure of phenotype. Clin. Pharmacol. Ther. 83, 234–242. 10.1038/sj.clpt.6100406 17971818

[B50] Gerth K. Kodidela S. Mahon M. Haque S. Verma N. Kumar S. (2019). Circulating extracellular vesicles containing xenobiotic metabolizing CYP enzymes and their potential roles in extrahepatic cells via cell-cell interactions. Int. J. Mol. Sci. 20, 6178. 10.3390/ijms20246178 31817878 PMC6940889

[B51] Gilardi G. Meharenna Y. T. Tsotsou G. E. Sadeghi S. J. Fairhead M. Giannini S. (2002). Molecular Lego: design of molecular assemblies of P450 enzymes for nanobiotechnology. Biosens. Bioelectron. 17, 133–145. 10.1016/s0956-5663(01)00286-x 11742744

[B52] Ginn S. L. Amaya A. K. Alexander I. E. Edelstein M. Abedi M. R. (2018). Gene therapy clinical trials worldwide to 2017: an update. J. Gene Med. 20, e3015. 10.1002/jgm.3015 29575374

[B53] Giuriato D. Correddu D. Catucci G. Di Nardo G. Bolchi C. Pallavicini M. (2022). Design of a H2O2‐generating P450SPα fusion protein for high yield fatty acid conversion. Protein Sci. 31, e4501. 10.1002/pro.4501 36334042 PMC9679977

[B54] Goetz M. P. Rae J. M. Suman V. J. Safgren S. L. Ames M. M. Visscher D. W. (2005). Pharmacogenetics of tamoxifen biotransformation is associated with clinical outcomes of efficacy and hot flashes. J. Clin. Oncol. 23, 9312–9318. 10.1200/JCO.2005.03.3266 16361630

[B55] Goh L. L. Lim C. W. Sim W. C. Toh L. X. Leong K. P. (2017). Analysis of genetic variation in CYP450 genes for clinical implementation. PLoS ONE 12, e0169233. 10.1371/journal.pone.0169233 28046094 PMC5207784

[B56] Goldwaser E. Laurent C. Lagarde N. Fabrega S. Nay L. Villoutreix B. O. (2022). Machine learning-driven identification of drugs inhibiting cytochrome P450 2C9. PLoS Comput. Biol. 18, e1009820. 10.1371/journal.pcbi.1009820 35081108 PMC8820617

[B57] Gomez A. Nekvindova J. Travica S. Lee M.-Y. Johansson I. Edler D. (2010). Colorectal cancer-specific cytochrome P450 2W1: intracellular localization, glycosylation, and catalytic activity. Mol. Pharmacol. 78, 1004–1011. 10.1124/mol.110.067652 20805301

[B58] Grohmann M. Paulmann N. Fleischhauer S. Vowinckel J. Priller J. Walther D. J. (2009). A mammalianized synthetic nitroreductase gene for high-level expression. BMC Cancer 9, 301. 10.1186/1471-2407-9-301 19712451 PMC3087338

[B59] Guengerich F. P. (2010). “Cytochrome P450 enzymes,” in Comprehensive toxicology. Editor McQueen C. A. (Oxford: Elsevier), 41–76. 10.1016/B978-0-08-046884-6.00404-8

[B60] Guengerich F. P. (2021). A history of the roles of cytochrome P450 enzymes in the toxicity of drugs. Toxicol. Res. 37, 1–23. 10.1007/s43188-020-00056-z 32837681 PMC7431904

[B61] Guengerich F. P. (2023). Drug metabolism: a half-century plus of progress, continued needs, and new opportunities. Drug Metab. Dispos. 51, 99–104. 10.1124/dmd.121.000739 35868640 PMC11024512

[B62] Gumulya Y. Baek J.-M. Wun S.-J. Thomson R. E. S. Harris K. L. Hunter D. J. B. (2018). Engineering highly functional thermostable proteins using ancestral sequence reconstruction. Nat. Catal. 1, 878–888. 10.1038/s41929-018-0159-5

[B63] Günther M. Waxman D. J. Wagner E. Ogris M. (2006). Effects of hypoxia and limited diffusion in tumor cell microenvironment on bystander effect of P450 prodrug therapy. Cancer Gene Ther. 13, 771–779. 10.1038/sj.cgt.7700955 16543915

[B64] Günzburg W. H. Salmons B. (2005). Use of cell therapy as a means of targeting chemotherapy to inoperable pancreatic cancer. Acta Biochim. Pol. 52, 601–607. 10.18388/abp.2005_3420 16175235

[B65] Hardiyanti Oktavia F. A. R. Nguyen N. A. Park C. M. Cha G. S. Nguyen T. H. H. Yun C.-H. (2023). CYP102A1 peroxygenase catalyzed reaction via *in situ* H2O2 generation. J. Inorg. Biochem. 242, 112165. 10.1016/j.jinorgbio.2023.112165 36848686

[B66] Harris K. L. Thomson R. E. S. Gumulya Y. Foley G. Carrera-Pacheco S. E. Syed P. (2022). Ancestral sequence reconstruction of a cytochrome P450 family involved in chemical defense reveals the functional evolution of a promiscuous, xenobiotic-metabolizing enzyme in vertebrates. Mol. Biol. Evol. 39, msac116. 10.1093/molbev/msac116 35639613 PMC9185370

[B67] Harris K. L. Thomson R. E. S. Strohmaier S. J. Gumulya Y. Gillam E. M. J. (2018). Determinants of thermostability in the cytochrome P450 fold. Biochim. Biophys. Acta Proteins Proteom 1866, 97–115. 10.1016/j.bbapap.2017.08.003 28822812

[B68] Hasegawa K. Koyama M. Funatsu K. (2010). Quantitative prediction of regioselectivity toward cytochrome P450/3A4 using machine learning approaches. Mol. Inf. 29, 243–249. 10.1002/minf.200900086 27462767

[B69] Hedley D. Ogilvie L. Springer C. (2007). Carboxypeptidase-G2-based gene-directed enzyme-prodrug therapy: a new weapon in the GDEPT armoury. Nat. Rev. Cancer 7, 870–879. 10.1038/nrc2247 17943135

[B70] Hernando-Rodriguez M. Rey-Barja N. Marichalar-Mendia X. Rodriguez-Tojo M. J. Acha-Sagredo A. Aguirre-Urizar J. M. (2012). Role of cytochrome P-450 genetic polymorphisms in oral carcinogenesis. J. Oral Pathol. Med. 41, 1–8. 10.1111/j.1600-0714.2011.01067.x 21793938

[B71] Huang Z. Raychowdhury M. K. Waxman D. J. (2000a). Impact of liver P450 reductase suppression on cyclophosphamide activation, pharmacokinetics and antitumoral activity in a cytochrome P450-based cancer gene therapy model. Cancer Gene Ther. 7, 1034–1042. 10.1038/sj.cgt.7700200 10917206

[B72] Huang Z. Roy P. Waxman D. J. (2000b). Role of human liver microsomal CYP3A4 and CYP2B6 in catalyzing N-dechloroethylation of cyclophosphamide and ifosfamide. Biochem. Pharmacol. 59, 961–972. 10.1016/s0006-2952(99)00410-4 10692561

[B73] Hunt S. (2001). Technology evaluation: MetXia-P450, oxford biomedica. Curr. Opin. Mol. Ther. 3, 595–598. PMID: 11804275. 11804275

[B74] Husain S. R. Han J. Au P. Shannon K. Puri R. K. (2015). Gene therapy for cancer: regulatory considerations for approval. Cancer Gene Ther. 22, 554–563. 10.1038/cgt.2015.58 26584531 PMC4722245

[B75] Hwang S. H. Hayashi K. Takayama K. Maitani Y. (2001). Liver-targeted gene transfer into a human hepatoblastoma cell line and *in vivo* by sterylglucoside-containing cationic liposomes. Gene Ther. 8, 1276–1280. 10.1038/sj.gt.3301510 11509962

[B76] Ja B. H C. J M. L S. S M. T C. (2016). A synthetic biology rheoswitch therapeutic System® for the controlled local expression of IL-12 as an immunotherapy for the treatment of cancer. Cell Biol. (Henderson, NV) 5. 10.4172/2324-9293.1000126

[B77] Jeffreys L. N. Girvan H. M. McLean K. J. Munro A. W. (2018). Characterization of cytochrome P450 enzymes and their applications in synthetic biology. Meth. Enzymol. 608, 189–261. 10.1016/bs.mie.2018.06.013 30173763

[B78] Jiang L. Huang L. Cai J. Xu Z. Lian J. (2021). Functional expression of eukaryotic cytochrome P450s in yeast. Biotechnol. Bioeng. 118, 1050–1065. 10.1002/bit.27630 33205834

[B79] Jounaidi Y. Chen C.-S. Veal G. J. Waxman D. J. (2006). Enhanced antitumor activity of P450 prodrug-based gene therapy using the low Km cyclophosphamide 4-hydroxylase P450 2B11. Mol. Cancer Ther. 5, 541–555. 10.1158/1535-7163.MCT-05-0321 16546968

[B80] Jounaidi Y. Waxman D. J. (2004). Use of replication-conditional adenovirus as a helper system to enhance delivery of P450 prodrug-activation genes for cancer therapy. Cancer Res. 64, 292–303. 10.1158/0008-5472.can-03-1798 14729637

[B81] Kalinichenko S. V. Shepelev M. V. Vikhreva P. N. Korobko I. V. (2017). A novel hybrid promoter ARE-hTERT for cancer gene therapy. Acta Naturae 9, 66–73. PMID: 29340219; PMCID: PMC5762830. 10.32607/2075851-2017-9-4-66-73 29340219 PMC5762830

[B82] Kan O. Griffiths L. Baban D. Iqball S. Uden M. Spearman H. (2001). Direct retroviral delivery of human cytochrome P450 2B6 for gene-directed enzyme prodrug therapy of cancer. Cancer Gene Ther. 8, 473–482. 10.1038/sj.cgt.7700329 11498768

[B83] Karjoo Z. Chen X. Hatefi A. (2016). Progress and problems with the use of suicide genes for targeted cancer therapy. Adv. Drug Deliv. Rev. 99, 113–128. 10.1016/j.addr.2015.05.009 26004498 PMC4758904

[B84] Kassi M. Stoner P. (2019). Gene therapy in cancer and consideration for the initiation of these therapies in clinical practice. AOAMJ 1, 1–11. 10.18103/aoam.v1i2.18

[B85] Kawahira H. Matsushita K. Shiratori T. Shimizu T. Nabeya Y. Hayashi H. (2010). Viral shedding after p53 adenoviral gene therapy in 10 cases of esophageal cancer. Cancer Sci. 101, 289–291. 10.1111/j.1349-7006.2009.01381.x 20175784 PMC11159950

[B86] Kedzie K. M. Grimm S. W. Chen F. Halpert J. R. (1993). Hybrid enzymes for structure-function analysis of cytochrome P-450 2B11. Biochimica Biophysica Acta (BBA) - Protein Struct. Mol. Enzym. 1164, 124–132. 10.1016/0167-4838(93)90238-M 8329443

[B87] Kivistö K. T. Kroemer H. K. Eichelbaum M. (1995). The role of human cytochrome P450 enzymes in the metabolism of anticancer agents: implications for drug interactions. Br. J. Clin. Pharmacol. 40, 523–530. 10.1111/j.1365-2125.1995.tb05796.x 8703657 PMC1365206

[B88] Korobkova E. A. (2015). Effect of natural polyphenols on CYP metabolism: implications for diseases. Chem. Res. Toxicol. 28, 1359–1390. 10.1021/acs.chemrestox.5b00121 26042469

[B89] Kowalski J. P. McDonald M. G. Whittington D. Guttman M. Scian M. Girhard M. (2019). Structure-activity relationships for CYP4B1 bioactivation of 4-ipomeanol congeners: direct correlation between cytotoxicity and trapped reactive intermediates. Chem. Res. Toxicol. 32, 2488–2498. 10.1021/acs.chemrestox.9b00330 31799839

[B90] Kratz F. Müller I. A. Ryppa C. Warnecke A. (2008). Prodrug strategies in anticancer chemotherapy. ChemMedChem 3, 20–53. 10.1002/cmdc.200700159 17963208

[B91] Kumar S. (2010). Engineering cytochrome P450 biocatalysts for biotechnology, medicine and bioremediation. Expert Opin. Drug Metab. Toxicol. 6, 115–131. 10.1517/17425250903431040 20064075 PMC2811222

[B92] Kumar S. Chen C. S. Waxman D. J. Halpert J. R. (2005). Directed evolution of mammalian cytochrome P450 2B1: mutations outside of the active site enhance the metabolism of several substrates, including the anticancer prodrugs cyclophosphamide and ifosfamide. J. Biol. Chem. 280, 19569–19575. 10.1074/jbc.M500158200 15774478

[B93] Lautier T. Urban P. Loeper J. Jezequel L. Pompon D. Truan G. (2016). Ordered chimerogenesis applied to CYP2B P450 enzymes. Biochim. Biophys. Acta 1860, 1395–1403. 10.1016/j.bbagen.2016.03.028 27015760

[B94] Le Blanc G. A. Waxman D. J. (1989). Interaction of anticancer drugs with hepatic monooxygenase enzymes. Drug Metab. Rev. 20, 395–439. 10.3109/03602538909103550 2680389

[B95] Lengler J. Omann M. Düvier D. Holzmüller H. Gregor W. Salmons B. (2006). Cytochrome P450 reductase dependent inhibition of cytochrome P450 2B1 activity: implications for gene directed enzyme prodrug therapy. Biochem. Pharmacol. 72, 893–901. 10.1016/j.bcp.2006.06.012 16887103

[B96] Lewis B. C. Mackenzie P. I. Miners J. O. (2011). Application of homology modeling to generate CYP1A1 mutants with enhanced activation of the cancer chemotherapeutic prodrug dacarbazine. Mol. Pharmacol. 80, 879–888. 10.1124/mol.111.072124 21816953

[B97] Li L. Lu Z. Liu G. Tang Y. Li W. (2023). Machine learning models to predict cytochrome P450 2B6 inhibitors and substrates. Chem. Res. Toxicol. 36, 1332–1344. 10.1021/acs.chemrestox.3c00065 37437120

[B98] Li X. Guo M. Hou B. Zheng B. Wang Z. Huang M. (2021). CRISPR/Cas9 nanoeditor of double knockout large fragments of E6 and E7 oncogenes for reversing drugs resistance in cervical cancer. J. Nanobiotechnology 19, 231. 10.1186/s12951-021-00970-w 34353334 PMC8340365

[B99] Li Z. Jiang Y. Guengerich F. P. Ma L. Li S. Zhang W. (2020). Engineering cytochrome P450 enzyme systems for biomedical and biotechnological applications. J. Biol. Chem. 295, 833–849. 10.1074/jbc.REV119.008758 31811088 PMC6970918

[B100] Libutti S. K. (2019). Recording 25 years of progress in cancer gene therapy. Cancer Gene Ther. 26, 345–346. 10.1038/s41417-019-0121-y 31285540

[B101] Liu Q. Dai G. Wu Y. Zhang M. Yang M. Wang X. (2022b). iRGD-modified exosomes-delivered BCL6 siRNA inhibit the progression of diffuse large B-cell lymphoma. Front. Oncol. 12, 822805. 10.3389/fonc.2022.822805 35982974 PMC9378967

[B102] Liu T. Zhang G. Chen Y.-H. Chen Y. Liu X. Peng J. (2006). Tissue specific expression of suicide genes delivered by nanoparticles inhibits gastric carcinoma growth. Cancer Biol. Ther. 5, 1683–1690. 10.4161/cbt.5.12.3379 17224635

[B103] Liu X. Li F. Sun T. Guo J. Zhang X. Zheng X. (2022a). Three pairs of surrogate redox partners comparison for Class I cytochrome P450 enzyme activity reconstitution. Commun. Biol. 5, 791. 10.1038/s42003-022-03764-4 35933448 PMC9357085

[B104] Liu Z. Liu C. Li J. Yu C. Jiang Y. (2011). The attitude of oncology physicians and nurses to the acceptance of new drugs for gene therapy. J. Cancer Educ. 26, 248–253. 10.1007/s13187-010-0172-0 20957467

[B105] Löhr M. Kröger J.-C. Hoffmeyer A. Freund M. Günzburg W. H. (2003). Safety, feasibility and clinical benefit of localized chemotherapy using microencapsulated cells for inoperable pancreatic carcinoma in a phase I/II trial Research Article. Cancer Ther. 1, 121–131.

[B106] Luo B. Yan D. Yan H. Yuan J. (2021). Cytochrome P450: implications for human breast cancer (Review). Oncol. Lett. 22, 548. 10.3892/ol.2021.12809 34093769 PMC8170261

[B107] Mahato R. Tai W. Cheng K. (2011). Prodrugs for improving tumor targetability and efficiency. Adv. Drug Deliv. Rev. 63, 659–670. 10.1016/j.addr.2011.02.002 21333700 PMC3132824

[B108] Malecki M. Proczka R. Chorostowska-Wynimko J. Swoboda P. Delbani A. Pachecka J. (2010). Recombinant adeno-associated viruses (rAAV2) facilitate the intraperitoneal gene delivery to cancer cells. Oncol. Lett. 1, 177–180. 10.3892/ol_00000032 22966278 PMC3436482

[B109] Malekshah O. M. Chen X. Nomani A. Sarkar S. Hatefi A. (2016). Enzyme/prodrug systems for cancer gene therapy. Curr. Pharmacol. Rep. 2, 299–308. 10.1007/s40495-016-0073-y 28042530 PMC5193473

[B110] McErlane V. Yakkundi A. McCarthy H. O. Hughes C. M. Patterson L. H. Hirst D. G. (2005). A cytochrome P450 2B6 meditated gene therapy strategy to enhance the effects of radiation or cyclophosphamide when combined with the bioreductive drug AQ4N. J. Gene Med. 7, 851–859. 10.1002/jgm.728 15712360

[B111] McFadyen M. C. E. Melvin W. T. Murray G. I. (2004). Cytochrome P 450 enzymes: novel options for cancer therapeutics. Mol. Cancer Ther. 3, 363–371. 10.1158/1535-7163.363.3.3 15026557

[B112] McIntosh J. A. Farwell C. C. Arnold F. H. (2014). Expanding P450 catalytic reaction space through evolution and engineering. Curr. Opin. Chem. Biol. 19, 126–134. 10.1016/j.cbpa.2014.02.001 24658056 PMC4008644

[B113] McLean K. J. Luciakova D. Belcher J. Tee K. L. Munro A. W. (2015). Biological diversity of cytochrome P450 redox partner systems. Adv. Exp. Med. Biol. 851, 299–317. 10.1007/978-3-319-16009-2_11 26002740

[B114] Mercapide J. Rappa G. Anzanello F. King J. Fodstad O. Lorico A. (2010). Primary gene-engineered neural stem/progenitor cells demonstrate tumor-selective migration and antitumor effects in glioma. Int. J. Cancer 126, 1206–1215. 10.1002/ijc.24809 19653275

[B115] Mirabbasi S. A. Khalighi K. Wu Y. Walker S. Khalighi B. Fan W. (2017). CYP2C19 genetic variation and individualized clopidogrel prescription in a cardiology clinic. Intern. Med. Perspect. 7, 151–156. 10.1080/20009666.2017.1347475 PMC5538219 28808507

[B116] Mishra A. P. Chandra S. Tiwari R. Srivastava A. Tiwari G. (2018). Therapeutic potential of prodrugs towards targeted drug delivery. Open Med. Chem. J. 12, 111–123. 10.2174/1874104501812010111 30505359 PMC6210501

[B117] Miura M. Ito K. Hayashi M. Nakajima M. Tanaka T. Ogura S. (2015). The effect of 5-aminolevulinic acid on cytochrome P450-mediated prodrug activation. PLoS ONE 10, e0131793. 10.1371/journal.pone.0131793 26181717 PMC4504516

[B118] Najjar A. Karaman R. (2019). Successes, failures, and future prospects of prodrugs and their clinical impact. Expert Opin. Drug Discov. 14, 199–220. 10.1080/17460441.2019.1567487 30714428

[B119] Naso M. F. Tomkowicz B. Perry W. L. Strohl W. R. (2017). Adeno-associated virus (AAV) as a vector for gene therapy. BioDrugs 31, 317–334. 10.1007/s40259-017-0234-5 28669112 PMC5548848

[B120] Nath A. Zientek M. A. Burke B. J. Jiang Y. Atkins W. M. (2010). Quantifying and predicting the promiscuity and isoform specificity of small-molecule cytochrome P450 inhibitors. Drug Metab. Dispos. 38, 2195–2203. 10.1124/dmd.110.034645 20841376 PMC2993456

[B121] Neyshaburinezhad N. Ghasim H. Rouini M. Daali Y. Ardakani Y. H. (2021). Frequency of important CYP450 enzyme gene polymorphisms in the Iranian population in comparison with other major populations: a comprehensive review of the human data. J. Pers. Med. 11, 804. 10.3390/jpm11080804 34442448 PMC8401584

[B122] Nguyen T.-A. Tychopoulos M. Bichat F. Zimmermann C. Flinois J.-P. Diry M. (2008). Improvement of cyclophosphamide activation by CYP2B6 mutants: from *in silico* to *ex vivo* . Mol. Pharmacol. 73, 1122–1133. 10.1124/mol.107.042861 18212249

[B123] Obermiller P. S. Tait D. L. Holt J. T. (2000). Gene therapy for carcinoma of the breast: therapeutic genetic correction strategies. Breast Cancer Res. 2, 28–31. 10.1186/bcr26 11250690 PMC521211

[B124] Ono K. Hashimoto H. Katayama T. Ueda N. Nagahama K. (2021). Injectable biocatalytic nanocomposite hydrogel factories for focal enzyme-prodrug cancer therapy. Biomacromolecules 22, 4217–4227. 10.1021/acs.biomac.1c00778 34546743

[B125] Ortiz de Montellano P. R. (2013). Cytochrome P450-activated prodrugs. Future Med. Chem. 5, 213–228. 10.4155/fmc.12.197 23360144 PMC3697796

[B126] Otey C. R. Silberg J. J. Voigt C. A. Endelman J. B. Bandara G. Arnold F. H. (2004). Functional evolution and structural conservation in chimeric cytochromes p450: calibrating a structure-guided approach. Chem. Biol. 11, 309–318. 10.1016/j.chembiol.2004.02.018 15123260

[B127] Ozdemir V. Gunes A. Dahl M.-L. Scordo M. G. Williams-Jones B. Someya T. (2006). Could endogenous substrates of drug-metabolizing enzymes influence constitutive physiology and drug target responsiveness? Pharmacogenomics 7, 1199–1210. 10.2217/14622416.7.8.1199 17184207

[B128] Parkinson O. T. Kelly E. J. Bezabih E. Whittington D. Rettie A. E. (2012). Bioactivation of 4-Ipomeanol by a CYP4B enzyme in bovine lung and inhibition by HET0016. J. Vet. Pharmacol. Ther. 35, 402–405. 10.1111/j.1365-2885.2011.01339.x 21919916 PMC3423095

[B129] Patterson L. H. Murray G. I. (2002). Tumour cytochrome P450 and drug activation. Curr. Pharm. Des. 8, 1335–1347. 10.2174/1381612023394502 12052211

[B130] Paul C. E. Churakova E. Maurits E. Girhard M. Urlacher V. B. Hollmann F. (2014). *In situ* formation of H2O2 for P450 peroxygenases. Bioorg. Med. Chem. 22, 5692–5696. 10.1016/j.bmc.2014.05.074 24984939

[B131] Pidkovka N. Rachkevych O. Belkhiri A. (2021). Extrahepatic cytochrome P450 epoxygenases: pathophysiology and clinical significance in human gastrointestinal cancers. Oncotarget 12, 379–391. 10.18632/oncotarget.27893 33659048 PMC7899545

[B132] Poltronieri P. D’Urso P. I. Mezzolla V. D’Urso O. F. (2013). Potential of anti-cancer therapy based on anti-miR-155 oligonucleotides in glioma and brain tumours. Chem. Biol. Drug Des. 81, 79–84. 10.1111/cbdd.12002 22834637

[B133] Preissner S. C. Hoffmann M. F. Preissner R. Dunkel M. Gewiess A. Preissner S. (2013). Polymorphic cytochrome P450 enzymes (CYPs) and their role in personalized therapy. PLoS ONE 8, e82562. 10.1371/journal.pone.0082562 24340040 PMC3858335

[B134] Quester K. Juarez-Moreno K. Secundino I. Roseinstein Y. Alejo K. P. Huerta-Saquero A. (2017). Cytochrome P450 bioconjugate as a nanovehicle for improved chemotherapy treatment. Macromol. Biosci. 17. 10.1002/mabi.201600374 27892656

[B135] Quiñones S. L. Rosero P. M. Roco A. Á. Moreno T. I. Sasso A. J. Varela F. N. (2008). Papel de las enzimas citocromo p450 en el metabolismo de fármacos antineoplásicos: Situación actual y perspectivas terapéuticas. Rev. Méd. Chile 136. 10.4067/S0034-98872008001000015 19194632

[B136] Rao D. (2023). The urgent need for healthcare workforce upskilling and ethical considerations in the era of AI-assisted medicine. Indian J. Otolaryngol. Head. Neck Surg. 75, 2638–2639. 10.1007/s12070-023-03755-9 PMC10132410 37362116

[B137] Reid J. M. Kuffel M. J. Miller J. K. Rios R. Ames M. M. (1999). Metabolic activation of dacarbazine by human cytochromes P450: the role of CYP1A1, CYP1A2, and CYP2E1. Clin. Cancer Res. 5, 2192–2197. 10473105

[B138] Reinen J. van Hemert D. Vermeulen N. P. E. Commandeur J. N. M. (2015). Application of a continuous-flow bioassay to investigate the organic solvent tolerability of cytochrome P450 BM3 mutants. J. Biomol. Screen. 20, 1246–1255. 10.1177/1087057115607183 26396180

[B139] Rendic S. Guengerich F. P. (2015). Survey of human oxidoreductases and cytochrome P450 enzymes involved in the metabolism of xenobiotic and natural chemicals. Chem. Res. Toxicol. 28, 38–42. 10.1021/tx500444e 25485457 PMC4303333

[B140] Rendic S. P. Guengerich F. P. (2021). Human Family 1-4 cytochrome P450 enzymes involved in the metabolic activation of xenobiotic and physiological chemicals: an update. Arch. Toxicol. 95, 395–472. 10.1007/s00204-020-02971-4 33459808 PMC7880545

[B141] Robson T. Hirst D. G. (2003). Transcriptional targeting in cancer gene therapy. J. Biomed. Biotechnol. 2003, 110–137. 10.1155/S1110724303209074 12721516 PMC323956

[B142] Roellecke K. Jäger V. D. Gyurov V. H. Kowalski J. P. Mielke S. Rettie A. E. (2017). Ligand characterization of CYP4B1 isoforms modified for high-level expression in *Escherichia coli* and HepG2 cells. Protein Eng. Des. Sel. 30, 205–216. 10.1093/protein/gzw075 28073960 PMC5421619

[B143] Roy P. Yu L. J. Crespi C. L. Waxman D. J. (1999). Development of a substrate-activity based approach to identify the major human liver P-450 catalysts of cyclophosphamide and ifosfamide activation based on cDNA-expressed activities and liver microsomal P-450 profiles. Drug Metab. Dispos. 27, 655–666. 10348794

[B144] Sadeghi S. J. Gilardi G. (2013). Chimeric P450 enzymes: activity of artificial redox fusions driven by different reductases for biotechnological applications. Biotechnol. Appl. Biochem. 60, 102–110. 10.1002/bab.1086 23586997

[B145] Salmons B. Löhr M. Günzburg W. H. (2003). Treatment of inoperable pancreatic carcinoma using a cell-based local chemotherapy: results of a phase I/II clinical trial. J. Gastroenterol. 38 (Suppl. 15), 78–84. 12698877

[B146] Schenkman J. B. Jansson I. (2006). Spectral analyses of cytochromes P450. Methods Mol. Biol. 320, 11–18. 10.1385/1-59259-998-2:11 16719370

[B147] Sellner M. Fischer A. Don C. G. Smieško M. (2021). Conformational landscape of cytochrome P450 reductase interactions. Int. J. Mol. Sci. 22, 1023. 10.3390/ijms22031023 33498551 PMC7864194

[B148] Seredina T. A. Goreva O. B. Talaban V. O. Grishanova A. Y. Lyakhovich V. V. (2012). Association of cytochrome P450 genetic polymorphisms with neoadjuvant chemotherapy efficacy in breast cancer patients. BMC Med. Genet. 13, 45. 10.1186/1471-2350-13-45 22702493 PMC3458973

[B149] Serrano D. Lazzeroni M. Zambon C. F. Macis D. Maisonneuve P. Johansson H. (2011). Efficacy of tamoxifen based on cytochrome P450 2D6, CYP2C19 and SULT1A1 genotype in the Italian Tamoxifen Prevention Trial. Pharmacogenomics J. 11, 100–107. 10.1038/tpj.2010.17 20309015

[B150] Seth P. (2005). Vector-mediated cancer gene therapy: an overview. Cancer Biol. Ther. 4, 512–517. 10.4161/cbt.4.5.1705 15908802

[B151] Shaw A. R. Suzuki M. (2019). Immunology of adenoviral vectors in cancer therapy. Mol. Ther. Methods Clin. Dev. 15, 418–429. 10.1016/j.omtm.2019.11.001 31890734 PMC6909129

[B152] Shinde M. B. Kajale Dr. A. D. Channawar Dr. M. A. Gawande Dr. S. R. (2020). Vector-mediated cancer gene therapy: a review. GSC Biol. Pharm. Sci. 13, 152–165. 10.30574/gscbps.2020.13.2.0368

[B153] Shoji O. Fujishiro T. Nishio K. Kano Y. Kimoto H. Chien S.-C. (2016). A substrate-binding-state mimic of H2 O2 -dependent cytochrome P450 produced by one-point mutagenesis and peroxygenation of non-native substrates. Catal. Sci. Technol. 6, 5806–5811. 10.1039/C6CY00630B

[B154] Smith P. W. Liu Y. Siefert S. A. Moskaluk C. A. Petroni G. R. Jones D. R. (2009). Breast cancer metastasis suppressor 1 (BRMS1) suppresses metastasis and correlates with improved patient survival in non-small cell lung cancer. Cancer Lett. 276, 196–203. 10.1016/j.canlet.2008.11.024 19111386 PMC4793277

[B155] Spence M. A. Kaczmarski J. A. Saunders J. W. Jackson C. J. (2021). Ancestral sequence reconstruction for protein engineers. Curr. Opin. Struct. Biol. 69, 131–141. 10.1016/j.sbi.2021.04.001 34023793

[B156] Squassina A. Manchia M. Mitropoulou C. Patrinos G. P. (2015). “An introduction to pharmacogenomics and personalized medicine,” in PanVascular medicine. Editor Lanzer P. (Berlin, Heidelberg: Springer Berlin Heidelberg), 1053–1065. 10.1007/978-3-642-37078-6_226

[B157] Steffens S. Frank S. Fischer U. Heuser C. Meyer K. L. Dobberstein K. U. (2000). Enhanced green fluorescent protein fusion proteins of herpes simplex virus type 1 thymidine kinase and cytochrome P450 4B1: applications for prodrug-activating gene therapy. Cancer Gene Ther. 7, 806–812. 10.1038/sj.cgt.7700173 10830728

[B158] Stipp M. C. Acco A. (2021). Involvement of cytochrome P450 enzymes in inflammation and cancer: a review. Cancer Chemother. Pharmacol. 87, 295–309. 10.1007/s00280-020-04181-2 33112969

[B159] Su J. Yu W. Gong M. You J. Liu J. Zheng J. (2015). Overexpression of a novel tumor metastasis suppressor gene TMSG1/LASS2 induces apoptosis via a caspase-dependent mitochondrial pathway. J. Cell. Biochem. 116, 1310–1317. 10.1002/jcb.25086 25735224

[B160] Sun I.-C. Yoon H. Y. Lim D.-K. Kim K. (2020). Recent trends in *in situ* enzyme-activatable prodrugs for targeted cancer therapy. Bioconjug. Chem. 31, 1012–1024. 10.1021/acs.bioconjchem.0c00082 32163277

[B161] Sun L. Chen C. S. Waxman D. J. Liu H. Halpert J. R. Kumar S. (2007). Re-engineering cytochrome P450 2B11dH for enhanced metabolism of several substrates including the anti-cancer prodrugs cyclophosphamide and ifosfamide. Arch. Biochem. Biophys. 458, 167–174. 10.1016/j.abb.2006.12.021 17254539 PMC1805465

[B162] Sung H. Ferlay J. Siegel R. L. Laversanne M. Soerjomataram I. Jemal A. (2021). Global cancer statistics 2020: GLOBOCAN estimates of incidence and mortality worldwide for 36 cancers in 185 countries. CA Cancer J. Clin. 71, 209–249. 10.3322/caac.21660 33538338

[B163] Sung Y. K. Kim S. W. (2019). Recent advances in the development of gene delivery systems. Biomater. Res. 23, 8. 10.1186/s40824-019-0156-z 30915230 PMC6417261

[B164] Szklarz G. D. He Y. Q. Kedzie K. M. Halpert J. R. Burnett V. L. (1996). Elucidation of amino acid residues critical for unique activities of rabbit cytochrome P450 2B5 using hybrid enzymes and reciprocal site-directed mutagenesis with rabbit cytochrome P450 2B4. Arch. Biochem. Biophys. 327, 308–318. 10.1006/abbi.1996.0127 8619620

[B165] Tapia-Moreno A. Juarez-Moreno K. Gonzalez-Davis O. Cadena-Nava R. D. Vazquez-Duhalt R. (2017). Biocatalytic virus capsid as nanovehicle for enzymatic activation of Tamoxifen in tumor cells. Biotechnol. J. 12. 10.1002/biot.201600706 28371407

[B166] Tatiparti K. Sau S. Kashaw S. K. Iyer A. K. (2017). siRNA delivery strategies: a comprehensive review of recent developments. Nanomater. (Basel) 7, 77. 10.3390/nano7040077 PMC5408169 28379201

[B167] Thoidingjam S. Sriramulu S. Freytag S. Brown S. L. Kim J. H. Chetty I. J. (2023). Oncolytic virus-based suicide gene therapy for cancer treatment: a perspective of the clinical trials conducted at Henry Ford Health. Transl. Med. Commun. 8, 11. 10.1186/s41231-023-00144-w 37065938 PMC10088621

[B168] Thomson R. E. S. Carrera-Pacheco S. E. Gillam E. M. J. (2022). Engineering functional thermostable proteins using ancestral sequence reconstruction. J. Biol. Chem. 298, 102435. 10.1016/j.jbc.2022.102435 36041629 PMC9525910

[B169] Touati W. Tran T. Seguin J. Diry M. Flinois J.-P. Baillou C. (2014). A suicide gene therapy combining the improvement of cyclophosphamide tumor cytotoxicity and the development of an anti-tumor immune response. Curr. Gene Ther. 14, 236–246. 10.2174/1566523214666140424152734 24766134

[B170] Travica S. Pors K. Loadman P. M. Shnyder S. D. Johansson I. Alandas M. N. (2013). Colon cancer-specific cytochrome P450 2W1 converts duocarmycin analogues into potent tumor cytotoxins. Clin. Cancer Res. 19, 2952–2961. 10.1158/1078-0432.CCR-13-0238 23589180

[B171] Tychopoulos M. Corcos L. Genne P. Beaune P. de Waziers I. (2005). A virus-directed enzyme prodrug therapy (VDEPT) strategy for lung cancer using a CYP2B6/NADPH-cytochrome P450 reductase fusion protein. Cancer Gene Ther. 12, 497–508. 10.1038/sj.cgt.7700817 15746946

[B172] Urban P. Lautier T. Pompon D. Truan G. (2018). Ligand access channels in cytochrome P450 enzymes: a review. Int. J. Mol. Sci. 19, 1617. 10.3390/ijms19061617 29848998 PMC6032366

[B173] van der Lee M. Allard W. G. Vossen R. H. A. M. Baak-Pablo R. F. Menafra R. Deiman B. A. L. M. (2020). A unifying model to predict variable drug response for personalised medicine. BioRxiv. 10.1101/2020.03.02.967554

[B174] van Vugt-Lussenburg B. M. A. Stjernschantz E. Lastdrager J. Oostenbrink C. Vermeulen N. P. E. Commandeur J. N. M. (2007). Identification of critical residues in novel drug metabolizing mutants of cytochrome P450 BM3 using random mutagenesis. J. Med. Chem. 50, 455–461. 10.1021/jm0609061 17266197

[B175] Verma H. Singh Bahia M. Choudhary S. Kumar Singh P. Silakari O. (2019). Drug metabolizing enzymes-associated chemo resistance and strategies to overcome it. Drug Metab. Rev. 51, 196–223. 10.1080/03602532.2019.1632886 31203662

[B176] Vredenburg G. den Braver-Sewradj S. van Vugt-Lussenburg B. M. A. Vermeulen N. P. E. Commandeur J. N. M. Vos J. C. (2015). Activation of the anticancer drugs cyclophosphamide and ifosfamide by cytochrome P450 BM3 mutants. Toxicol. Lett. 232, 182–192. 10.1016/j.toxlet.2014.11.005 25448283

[B177] Wang D. Tai P. W. L. Gao G. (2019). Adeno-associated virus vector as a platform for gene therapy delivery. Nat. Rev. Drug Discov. 18, 358–378. 10.1038/s41573-019-0012-9 30710128 PMC6927556

[B178] Wang N.-N. Wang X.-G. Xiong G.-L. Yang Z.-Y. Lu A.-P. Chen X. (2022). Machine learning to predict metabolic drug interactions related to cytochrome P450 isozymes. J. Cheminform. 14, 23. 10.1186/s13321-022-00602-x 35428354 PMC9013037

[B179] Wang X. Y. Martiniello-Wilks R. Shaw J. M. Ho T. Coulston N. Cooke-Yarborough C. (2004). Preclinical evaluation of a prostate-targeted gene-directed enzyme prodrug therapy delivered by ovine atadenovirus. Gene Ther. 11, 1559–1567. 10.1038/sj.gt.3302308 15343359

[B180] Wang Y. Yuan F. (2006). Delivery of viral vectors to tumor cells: extracellular transport, systemic distribution, and strategies for improvement. Ann. Biomed. Eng. 34, 114–127. 10.1007/s10439-005-9007-2 16520902

[B181] Waskell L. Kim J.-J. P. (2015). “Electron transfer partners of cytochrome P450,” in Cytochrome P450. Editor Ortiz de Montellano P. R. (Cham: Springer International Publishing), 33–68. 10.1007/978-3-319-12108-6_2

[B182] Waxman D. J. (1993). Activation of thio-tepa cytotoxicity toward human breast-cancer cells by hepatic cytochrome-P450. Int. J. Oncol. 2, 731–738. 10.3892/ijo.2.5.731 21573617

[B183] Waxman D. J. Chen L. Hecht J. E. Jounaidi Y. (1999). Cytochrome P450-based cancer gene therapy: recent advances and future prospects. Drug Metab. Rev. 31, 503–522. 10.1081/dmr-100101933 10335450

[B184] Wendel H.-G. de Stanchina E. Cepero E. Ray S. Emig M. Fridman J. S. (2006). Loss of p53 impedes the antileukemic response to BCR-ABL inhibition. Proc. Natl. Acad. Sci. U. S. A. 103, 7444–7449. 10.1073/pnas.0602402103 16651519 PMC1455409

[B185] Wiek C. Schmidt E. M. Roellecke K. Freund M. Nakano M. Kelly E. J. (2015). Identification of amino acid determinants in CYP4B1 for optimal catalytic processing of 4-ipomeanol. Biochem. J. 465, 103–114. 10.1042/BJ20140813 25247810 PMC4312185

[B186] Xiao R. Wang L. Carvalho L. Wassmer S. Vandenberghe L. H. (2016). 608. *in silico* reconstructed ancestral adeno-associated viruses transduce mouse anterior segment. Mol. Ther. 24, S241. 10.1016/S1525-0016(16)33416-5

[B187] Xu C. Li C. Y.-T. Kong A.-N. T. (2005). Induction of phase I, II and III drug metabolism/transport by xenobiotics. Arch. Pharm. Res. 28, 249–268. 10.1007/BF02977789 15832810

[B188] Yan W. Zhang W. Jiang T. (2011). Oncogene addiction in gliomas: implications for molecular targeted therapy. J. Exp. Clin. Cancer Res. 30, 58. 10.1186/1756-9966-30-58 21575270 PMC3113747

[B189] Zanger U. M. Schwab M. (2013). Cytochrome P450 enzymes in drug metabolism: regulation of gene expression, enzyme activities, and impact of genetic variation. Pharmacol. Ther. 138, 103–141. 10.1016/j.pharmthera.2012.12.007 23333322

[B190] Zhang J. Kale V. Chen M. (2015). Gene-directed enzyme prodrug therapy. AAPS J. 17, 102–110. 10.1208/s12248-014-9675-7 25338741 PMC4287286

[B191] Zhang K. El Damaty S. Fasan R. (2011). P450 fingerprinting method for rapid discovery of terpene hydroxylating P450 catalysts with diversified regioselectivity. J. Am. Chem. Soc. 133, 3242–3245. 10.1021/ja109590h 21341707

[B192] Zhang L. Wang Q. (2022). Harnessing P450 enzyme for biotechnology and synthetic biology. Chembiochem 23, e202100439. 10.1002/cbic.202100439 34542923

[B193] Zhang S. Lin Q. D. Di W. (2006). Suppression of human ovarian carcinoma metastasis by the metastasis-suppressor gene, BRMS1. Int. J. Gynecol. Cancer 16, 522–531. 10.1111/j.1525-1438.2006.00547.x 16681721

[B194] Zhang T. Zhao M. Pang Y. Zhang W. Angela Liu L. Wei D.-Q. (2012). Recent progress on bioinformatics, functional genomics, and metabolomics research of cytochrome P450 and its impact on drug discovery. Curr. Top. Med. Chem. 12, 1346–1355. 10.2174/156802612801319052 22690681

[B195] Zhao M. Ma J. Li M. Zhang Y. Jiang B. Zhao X. (2021). Cytochrome P450 enzymes and drug metabolism in humans. Int. J. Mol. Sci. 22, 12808. 10.3390/ijms222312808 34884615 PMC8657965

[B196] Zhao Z. Anselmo A. C. Mitragotri S. (2022). Viral vector-based gene therapies in the clinic. Bioeng. Transl. Med. 7, e10258. 10.1002/btm2.10258 35079633 PMC8780015

[B197] Zheng Y. i.-M. Henne K. R. Charmley P. Kim R. B. McCarver D. G. Cabacungan E. T. (2003). Genotyping and site-directed mutagenesis of a cytochrome P450 meander Pro-X-Arg motif critical to CYP4B1 catalysis. Toxicol. Appl. Pharmacol. 186, 119–126. 10.1016/S0041-008X(02)00028-5 12639503

[B198] Zhou S.-F. Liu J.-P. Chowbay B. (2009). Polymorphism of human cytochrome P450 enzymes and its clinical impact. Drug Metab. Rev. 41, 89–295. 10.1080/03602530902843483 19514967

[B199] Zinn E. Pacouret S. Khaychuk V. Turunen H. T. Carvalho L. S. Andres-Mateos E. (2015). *In silico* reconstruction of the viral evolutionary lineage yields a potent gene therapy vector. Cell Rep. 12, 1056–1068. 10.1016/j.celrep.2015.07.019 26235624 PMC4536165

[B200] Zorde Khvalevsky E. Gabai R. Rachmut I. H. Horwitz E. Brunschwig Z. Orbach A. (2013). Mutant KRAS is a druggable target for pancreatic cancer. Proc. Natl. Acad. Sci. U. S. A. 110, 20723–20728. 10.1073/pnas.1314307110 24297898 PMC3870687

[B201] Zu H. Gao D. (2021). Non-viral vectors in gene therapy: recent development, challenges, and prospects. AAPS J. 23, 78. 10.1208/s12248-021-00608-7 34076797 PMC8171234

