# Supplementary material for: Designing cytochrome P450 enzymes for use in cancer gene therapy
Source: Front Bioeng Biotechnol. 2024 May 24;12:1405466. doi: 10.3389/fbioe.2024.1405466 (PMC11164052; doi:10.3389/fbioe.2024.1405466)
Supplement: Supplementary file 1 [file Table1.docx]

| **CYP name** | **Mutations or changes** | **Reaction** | **Km (mM)** | **Vmax (mol/min/mol)** | **Vmax/Km (mol/min/mol P450/ mM)** | **Kcat (min^-1^)** | **Kcat/Km (min^-1^ mM^-1^)** | **SA (pmol product/min/mg enzyme)** | **K_D_ (**µM) | **Conver-**  **sion**  **(**µM) | **Prodrug** | **Approach** | **Ref** |
| --- | --- | --- | --- | --- | --- | --- | --- | --- | --- | --- | --- | --- | --- |
| 2B1 | WT | 4-hydroxylation | 1.45 | 35.9 | 24.9 | N.D. | N.D. | N.D. | N.D. | N.D. | CPA | Rational/ Site-directed mutagenesis | [(Chen et al. 2004)](https://sciwheel.com/work/citation?ids=532772&pre=&suf=&sa=0) |
|  | I114V |  | 0.4 | 20 | 50 | N.D. | N.D. | N.D. | N.D. | N.D. |  |  |  |
|  | WT |  | 1.73 | 13.2 | 7.9 | N.D. | N.D. | N.D. | N.D. | N.D. | IFA |  |  |
|  | V363A |  | 1 | 10 | 9 | N.D. | N.D. | N.D. | N.D. | N.D. |  |  |  |
|  | 2B1dH (N-terminal modified) |  | ~0.4 | N.D. | N.D. | ~34 | ~75 | N.D. | N.D. | N.D. | CPA | Directed evolution | [(Kumar et al. 2005)](https://sciwheel.com/work/citation?ids=534628&pre=&suf=&sa=0) |
|  | L209A, S334P |  | ~0.2 | N.D. | N.D. | ~50 | ~220 | N.D. | N.D. | N.D. |  |  |  |
|  | 2B1dH (N-terminal modified) |  | ~0.5 | N.D. | N.D. | ~10 | ~22 | N.D. | N.D. | N.D. | IFA |  |  |
|  | L209A, V183L |  | ~0.3 | N.D. | N.D. | ~24 | ~70 | N.D. | N.D. | N.D. |  |  |  |
| 2B6 | WT |  | 4.9 | 62.5 | 12.8 | N.D. | N.D. | N.D. | N.D. | N.D. | CPA | Rational/ Site-directed mutagenesis, molecular dynamics | [(Nguyen et al. 2008)](https://sciwheel.com/work/citation?ids=535214&pre=&suf=&sa=0) |
|  | I114V/ V477W |  | 1.1 | 58.5 | 52.7 | N.D. | N.D. | N.D. | N.D. | N.D. |  |  |  |
| 2B6TM | L199M, I114V, V477W |  | 1.05 | 105.5 | 100.5 | N.D. | N.D. | N.D. | N.D. | N.D. | CPA | Rational/ Site-directed mutagenesis | [(Touati et al. 2014)](https://sciwheel.com/work/citation?ids=1701515&pre=&suf=&sa=0) |
| 2B11 | WT |  | 0.16 | 28.2 | 174.7 | 28 | 175 | N.D. | N.D. | N.D. | CPA | Rational/ Site-directed mutagenesis | [(Sun et al. 2007](https://sciwheel.com/work/citation?ids=15712894&pre=&suf=&sa=0);[Chen et al. 2004)](https://sciwheel.com/work/citation?ids=532772&pre=&suf=&sa=0) |
|  | 2B11dH V183L |  | 0.06 | N.D. | N.D. | ~24 | ~400 | N.D. | N.D. | N.D. |  |  |  |
|  | WT |  | 0.08 | 5.3 | 66.8 | 5.4 | 54 | N.D. | N.D. | N.D. | IFA |  |  |
|  | 2B11dH V183L |  | 0.03 | N.D. | N.D. | ~2.8 | ~93 | N.D. | N.D. | N.D. |  |  |  |
| 2B6 and 2B11 | 2B6 WT |  | 4.03 | N.D. | N.D. | N.D. | N.D. | 23.2 | N.D. | N.D. | CPA | Semi-rational/combinatorial approach of protein quantitative  structure–activity relationships (QSAR) | [(Lautier et al. 2016)](https://sciwheel.com/work/citation?ids=15875676&pre=&suf=&sa=0) |
|  | 2B11 WT |  | 0.08 | N.D. | N.D. | N.D. | N.D. | 13.2 | N.D. | N.D. |  |  |  |
|  | Chim K |  | 0.07 | N.D. | N.D. | N.D. | N.D. | 8.3 | N.D. | N.D. |  |  |  |
|  | Chim O |  | 0.02 | N.D. | N.D. | N.D. | N.D. | 11.8 | N.D. | N.D. |  |  |  |
| BM3 (CYP102A1) | R47L, E64G, F81I, F87V, E143G, L188Q, Y198C, E267V, H285Y, G415S **(M11)** |  | 0.087- 0.16 | 896- 1614 | 10315- 10078 | N.D. | N.D. | N.D. | N.D. | N.D. | CPA | Rational/ Site-directed mutagenesis and random mutagenesis | [(Vredenburg et al. 2015; van Vugt-Lussenburg et al. 2007; Damsten et al. 2008)](https://sciwheel.com/work/citation?ids=12975666,15740130,2592550&pre=&pre=&pre=&suf=&suf=&suf=&sa=0,0,0) |
|  | R47L, E64G, F81I, F87V, E143G, L188Q, Y198C, E267V, H285Y, G415S, L437S |  | 0.088- 0.115 | 757-938 | 8620- 8129 | N.D. | N.D. | N.D. | N.D. | N.D. | CPA |  |  |
|  | R47L, E64G, F81I, F87V, E143G, L188Q, Y198C, E267V, H285Y, G415S, L437S |  | N.D. | N.D. | 577- 580.9 | N.D. | N.D. | N.D. | N.D. | N.D. | IFA |  |  |
|  | M11 |  | N.D. | N.D. | 1306- 1430 | N.D. | N.D. | N.D. | N.D. | N.D. | IFA |  |  |
| 4B1 | WT | Furan ring epoxidation | N.D. | N.D. | N.D. | N.D. | N.D. | N.D. | 30 | 16.8 | 4-IPO |  |  |
|  | S427P, R124K, E130D, E159D, R199K, T202S, D217E, L135F, V156I, L226I, T158A, E170K, N190D **(hP427+12)** |  | N.D. | N.D. | N.D. | N.D. | N.D. | N.D. | 28 | 11.6 |  | Rational/Homology model, mutagenesis | [(Wiek et al. 2015)](https://sciwheel.com/work/citation?ids=349741&pre=&suf=&sa=0) |
|  | S427R |  | N.D. | N.D. | N.D. | N.D. | N.D. | N.D. | 8 | 5.1 |  | N.A. | [(Roellecke et al. 2017)](https://sciwheel.com/work/citation?ids=16292153&pre=&suf=&sa=0) |
|  | hP427+12 |  | N.D. | N.D. | N.D. | N.D. | N.D. | N.D. | 1.31 | 150.9 | PK |  |  |
|  | S427R |  | N.D. | N.D. | N.D. | N.D. | N.D. | N.D. | 0.25 | 42.3 |  |  |  |
| 1A1 | E161K | N-demethylation | 0.249 | 30 | 120.5 | N.D. | N.D. | N.D. | N.D. |  | DTIC | Rational/Homology model, mutagenesis | [(Lewis et al. 2011)](https://sciwheel.com/work/citation?ids=15772998&pre=&suf=&sa=0) |
|  | V228T |  | 0.386 | 32.4 | 83.9 | N.D. | N.D. | N.D. | N.D. |  |  |  |  |
|  | E256K |  | 0.238 | 29 | 121.8 | N.D. | N.D. | N.D. | N.D. |  |  |  |  |

CPA: cyclophosphamide; IFA: ifosfamde; 4-IPO: 4-ipomeanol; DTIC: dacarbazine; PK: perilla ketone; N.D.: Not determined; WT: wild type.
